# Genome-Wide Association Study of Blood Pressure Extremes Identifies Variant near *UMOD* Associated with Hypertension

**DOI:** 10.1371/journal.pgen.1001177

**Published:** 2010-10-28

**Authors:** Sandosh Padmanabhan, Olle Melander, Toby Johnson, Anna Maria Di Blasio, Wai K. Lee, Davide Gentilini, Claire E. Hastie, Cristina Menni, Maria Cristina Monti, Christian Delles, Stewart Laing, Barbara Corso, Gerjan Navis, Arjan J. Kwakernaak, Pim van der Harst, Murielle Bochud, Marc Maillard, Michel Burnier, Thomas Hedner, Sverre Kjeldsen, Björn Wahlstrand, Marketa Sjögren, Cristiano Fava, Martina Montagnana, Elisa Danese, Ole Torffvit, Bo Hedblad, Harold Snieder, John M. C. Connell, Morris Brown, Nilesh J. Samani, Martin Farrall, Giancarlo Cesana, Giuseppe Mancia, Stefano Signorini, Guido Grassi, Susana Eyheramendy, H. Erich Wichmann, Maris Laan, David P. Strachan, Peter Sever, Denis Colm Shields, Alice Stanton, Peter Vollenweider, Alexander Teumer, Henry Völzke, Rainer Rettig, Christopher Newton-Cheh, Pankaj Arora, Feng Zhang, Nicole Soranzo, Timothy D. Spector, Gavin Lucas, Sekar Kathiresan, David S. Siscovick, Jian'an Luan, Ruth J. F. Loos, Nicholas J. Wareham, Brenda W. Penninx, Ilja M. Nolte, Martin McBride, William H. Miller, Stuart A. Nicklin, Andrew H. Baker, Delyth Graham, Robert A. McDonald, Jill P. Pell, Naveed Sattar, Paul Welsh, Global BPgen Consortium, Patricia Munroe, Mark J. Caulfield, Alberto Zanchetti, Anna F. Dominiczak

**Affiliations:** 1 Institute of Cardiovascular and Medical Sciences, College of Medical Veterinary and Life Sciences, University of Glasgow, Glasgow, United Kingdom; 2 Department of Clinical Sciences, Hypertension and Cardiovascular Diseases, University Hospital Malmö, Lund University, Malmö, Sweden; 3 Clinical Pharmacology and Barts and the London Genome Centre, William Harvey Research Institute, Barts and the London School of Medicine, Queen Mary University of London, London, United Kingdom; 4 Istituto Auxologico Italiano, Milan, Italy; 5 Università Milano-Bicocca, Dipartimento di Medicina Clinica e Prevenzione, Ospedale San Gerardo, Monza, Milano, Italy; 6 Department of Health Science, University of Pavia, Pavia, Italy; 7 Department of Nephrology, University Medical Center Groningen, University of Groningen, Groningen, The Netherlands; 8 Department of Cardiology, University Medical Center Groningen, University of Groningen, Groningen, The Netherlands; 9 University Institute of Social and Preventive Medicine, Centre Hospitalier Universitaire Vaudois and University of Lausanne, Lausanne, Switzerland; 10 Service of Nephrology, Centre Hospitalier Universitaire Vaudois and University of Lausanne, Lausanne, Switzerland; 11 Institute of Medicine, The Sahlgrenska Academy, University of Gothenburg, Gothenburg, Sweden; 12 Department of Cardiology, University of Oslo, Ullevaal Hospital, Oslo, Norway; 13 Department of Medicine, Section of Internal Medicine C, University of Verona, Verona, Italy; 14 Department of Life and Reproduction Sciences, Section of Clinical Chemistry, University of Verona, Verona, Italy; 15 Department of Nephrology, Institution of Clinical Sciences, University Hospital of Lund, Lund, Sweden; 16 Unit of Genetic Epidemiology and Bioinformatics, Department of Epidemiology, University Medical Center Groningen, University of Groningen, Groningen, The Netherlands; 17 College of Medicine, Dentistry and Nursing, Ninewells Hospital, University of Dundee, Dundee, United Kingdom; 18 Clinical Pharmacology Unit, University of Cambridge, Addenbrookes Hospital, Cambridge, United Kingdom; 19 Department of Cardiovascular Sciences, University of Leicester, Glenfield Hospital, Leicester, United Kingdom; 20 Department of Cardiovascular Medicine, Wellcome Trust Centre for Human Genetics, Oxford, United Kingdom; 21 Azienda Ospedaliera di Desio e Vimercate, Milano, Italy; 22 Department of Statistics, Pontificia Universidad Catolica de Chile, Santiago, Chile; 23 Institute of Epidemiology, Helmholtz Zentrum München, German Research Center for Environmental Health, Neuherberg, Germany; 24 Institute of Medical Informatics, Biometry and Epidemiology, Chair of Epidemiology, Ludwig-Maximilians-Universität, Munich, Germany; 25 Institute of Molecular and Cell Biology, University of Tartu, Tartu, Estonia; 26 Division of Community Health Sciences, St George's, University of London, London, United Kingdom; 27 International Centre for Circulatory Health National Heart and Lung Institute, Imperial College, London, United Kingdom; 28 Conway Institute of Biomolecular and Biomedical Research, University College Dublin, Dublin, Ireland; 29 Molecular and Cellular Therapeutics, Royal College of Surgeons in Ireland, Dublin, Ireland; 30 Department of Internal Medicine, Centre Hospitalier Universitaire Vaudois and University of Lausanne, Lausanne, Switzerland; 31 Interfaculty Institute for Genetics and Functional Genomics, University of Greifswald, Greifswald, Germany; 32 Institute for Community Medicine, University of Greifswald, Greifswald, Germany; 33 Institute of Physiology, University of Greifswald, Greifswald, Germany; 34 Center for Human Genetic Research and Cardiovascular Research Center, Massachusetts General Hospital, Harvard Medical School, Boston, Massachusetts, United States of America; 35 Program in Medical and Population Genetics, Broad Institute, Cambridge, Massachusetts, United States of America; 36 Department of Twin Research and Genetic Epidemiology, King's College London, London, United Kingdom; 37 Wellcome Trust Sanger Institute, Genome Campus, Hinxton, United Kingdom; 38 Cardiovascular Epidemiology and Genetics Group, Institut Municipal d'Investigacio Medica, Barcelona, Spain; 39 Cardiovascular Health Research Unit, Departments of Medicine and Epidemiology, University of Washington, Seattle, Washington, United States of America; 40 MRC Epidemiology Unit, Institute of Metabolic Science, Cambridge, United Kingdom; 41 Department of Psychiatry/EMGO Institute, Neuroscience Campus, VU University Medical Center, Amsterdam, The Netherlands; 42 Department of Psychiatry, Leiden University Medical Center, Leiden, The Netherlands; 43 Department of Psychiatry, University Medical Center Groningen, University of Groningen, Groningen, The Netherlands; 44 Public Health and Health Policy Section, University of Glasgow, Glasgo, United Kingdom; 45 University of Milano, Milano, Italy; University of California San Diego and The Scripps Research Institute, United States of America

## Abstract

Hypertension is a heritable and major contributor to the global burden of disease. The sum of rare and common genetic variants robustly identified so far explain only 1%–2% of the population variation in BP and hypertension. This suggests the existence of more undiscovered common variants. We conducted a genome-wide association study in 1,621 hypertensive cases and 1,699 controls and follow-up validation analyses in 19,845 cases and 16,541 controls using an extreme case-control design. We identified a locus on chromosome 16 in the 5′ region of Uromodulin (*UMOD*; rs13333226, combined P value of 3.6×10^−11^). The minor G allele is associated with a lower risk of hypertension (OR [95%CI]: 0.87 [0.84–0.91]), reduced urinary uromodulin excretion, better renal function; and each copy of the G allele is associated with a 7.7% reduction in risk of CVD events after adjusting for age, sex, BMI, and smoking status (H.R. = 0.923, 95% CI 0.860–0.991; p = 0.027). In a subset of 13,446 individuals with estimated glomerular filtration rate (eGFR) measurements, we show that rs13333226 is independently associated with hypertension (unadjusted for eGFR: 0.89 [0.83–0.96], p = 0.004; after eGFR adjustment: 0.89 [0.83–0.96], p = 0.003). In clinical functional studies, we also consistently show the minor G allele is associated with lower urinary uromodulin excretion. The exclusive expression of uromodulin in the thick portion of the ascending limb of Henle suggests a putative role of this variant in hypertension through an effect on sodium homeostasis. The newly discovered *UMOD* locus for hypertension has the potential to give new insights into the role of uromodulin in BP regulation and to identify novel drugable targets for reducing cardiovascular risk.

## Author Summary

Hypertension is the leading contributor to global mortality with a global prevalence of 26.4% in 2000, projected to increase to 29.2% by 2025. While 50%–60% of population variation in blood pressure can be attributable to additive genetic factors, all the genetic variants robustly identified so far explain only 1%–2% of the population variance indicating the presence of additional undiscovered risk variants. Using an extreme case-control strategy, we have discovered a SNP in the promoter region of the uromodulin gene (*UMOD*) to be associated with hypertension (minor allele protective against hypertension). We then validated this association using large-scale population and case-control studies, where similar extreme criteria for selection of cases and controls have been used (21,466 cases and 18,240 controls). As the locus was related to uromodulin, a protein exclusively expressed in the kidneys, we show that the association is independent of renal dysfunction. We also show preliminary evidence that the SNP allele which is protective against hypertension is also protective against cardiovascular events in 26,654 Swedish subjects followed-up for 12 years. The newly discovered *UMOD* locus for hypertension has the potential to give unique insights into the role of uromodulin in BP regulation and to identify novel drugable targets.

## Introduction

Hypertension is a major cardiovascular risk factor with a global prevalence of 26.4% in 2000, projected to increase to 29.2% by 2025, and is the leading contributor to global mortality[1], [2]. While epidemiologically BP is a trait continuously associated with an increased risk of cardiovascular mortality and morbidity, clinical risk assessment is necessarily based on a predefined threshold at which the quantitative BP phenotype is converted into a binary trait (hypertension) [3]–[6]. The main justification for large scale efforts to determine the genetic underpinnings of BP regulation is to identify new pharmacological targets for BP reduction while advancing our understanding of blood pressure regulation. This in turn could lead to novel prevention strategies to reduce the growing public health burden of hypertension-related cardiovascular disease [2], [7]. Systemic blood pressure (BP) is determined primarily by cardiac output and total peripheral resistance, which are controlled by a complex network of interacting pathways involving renal, neural, endocrine, vascular and environmental factors. So far, the search for common variants affecting BP has identified thirteen loci from two large meta-analyses consortia, with each association explaining only a very small proportion of the total variation in systolic or diastolic blood pressure (SBP or DBP; ∼0.05–0.10%, approximately 1 mmHg per allele SBP or 0.5 mmHg per allele DBP)[8], [9]. The sum of rare and common genetic variants robustly identified so far through linkage and genome wide association studies explain only 1–2% of the population variation in BP and hypertension. These data suggest the existence of more undiscovered blood pressure related common variants. Cross-sectional studies of the general population have required extremely large sample sizes to detect such small effect sizes [10]. In this paper we explored an alternative strategy to increase power, using cases and controls drawn from the extremes of the BP distribution, and detected a novel locus associated with hypertension. We then validated this association using large-scale population and case-control studies, where similar extreme criteria for selection of cases and controls have been used. As the locus was related to uromodulin, a protein exclusively expressed intrarenally, we tested for dependency of the association on renal function (eGFR) and urinary excretion of uromodulin. Finally, we tested associations with cardiovascular outcomes.

## Results

### Genome-wide association, replication, and meta-analysis

**Figure 1:**
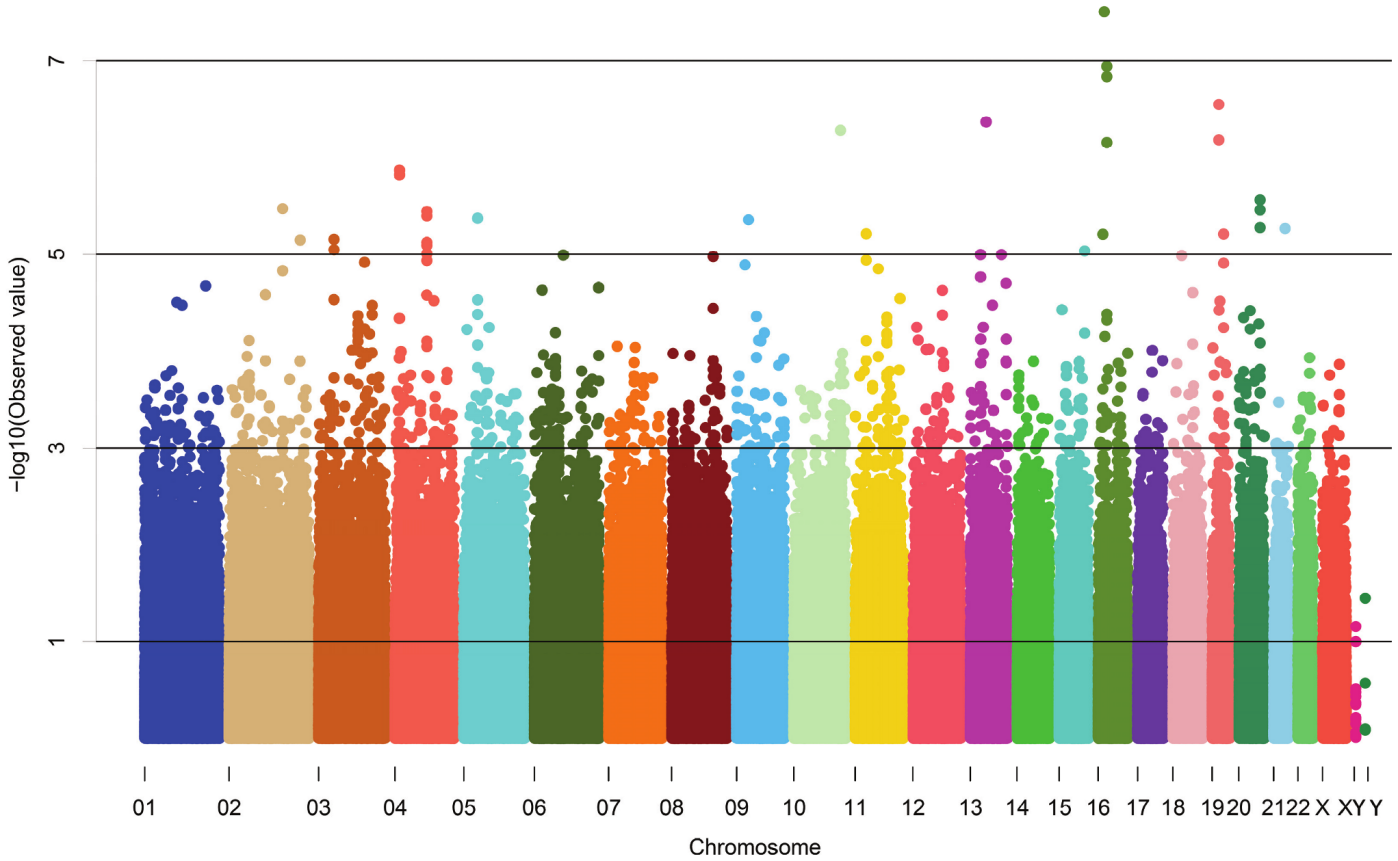
Manhattan plot of genomewide –log_10_(p-value) from association analysis of BP extremes in the discovery sample.

**Figure 2:**
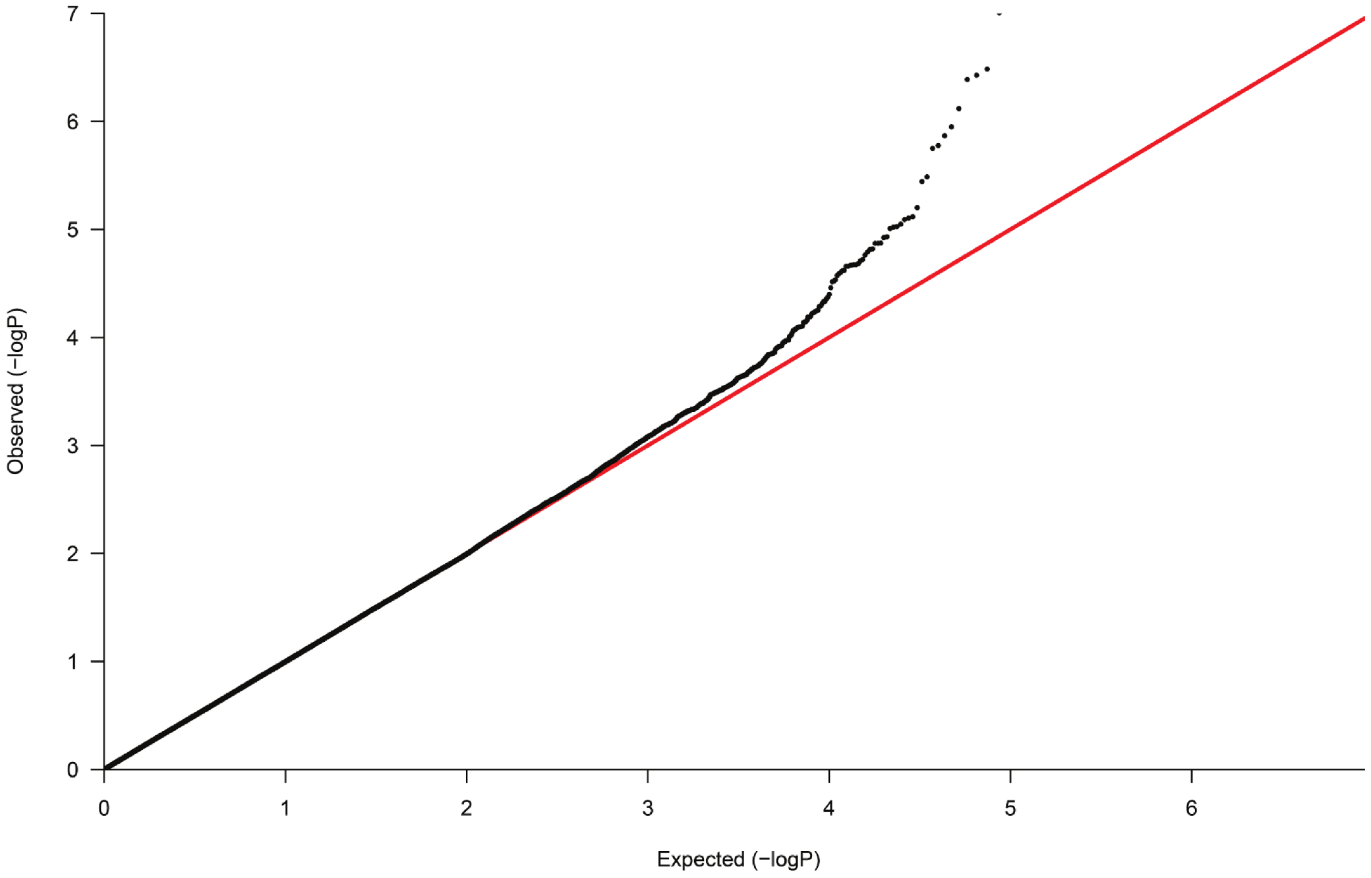
Quantile-Quantile plot of observed versus expected p-value distributions in the discovery sample.

**Figure 3:**
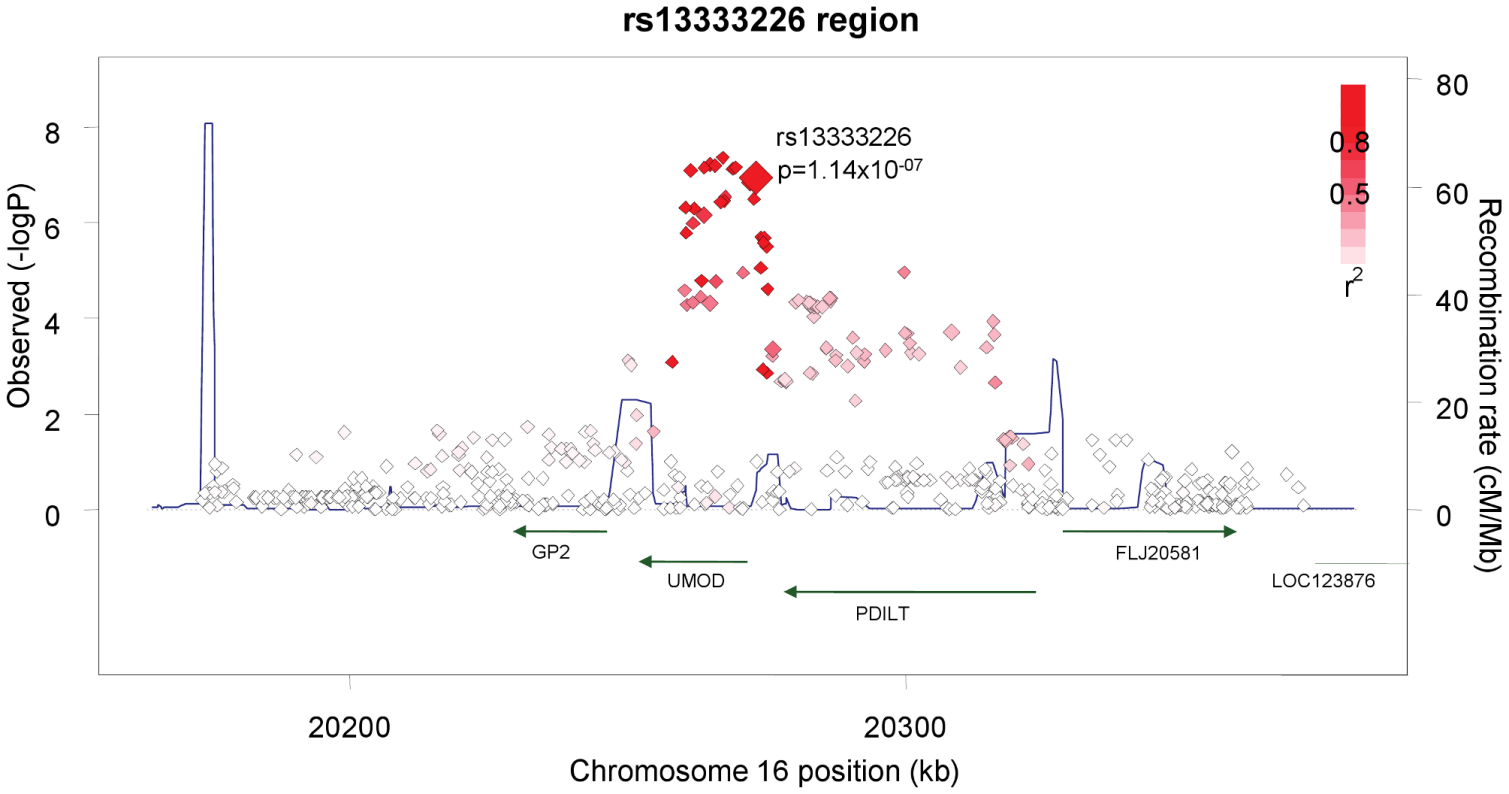
Association plot of the genomic region around rs13333226 showing both typed and imputed SNPs with location of genes and recombination rate.

**Figure 4:**
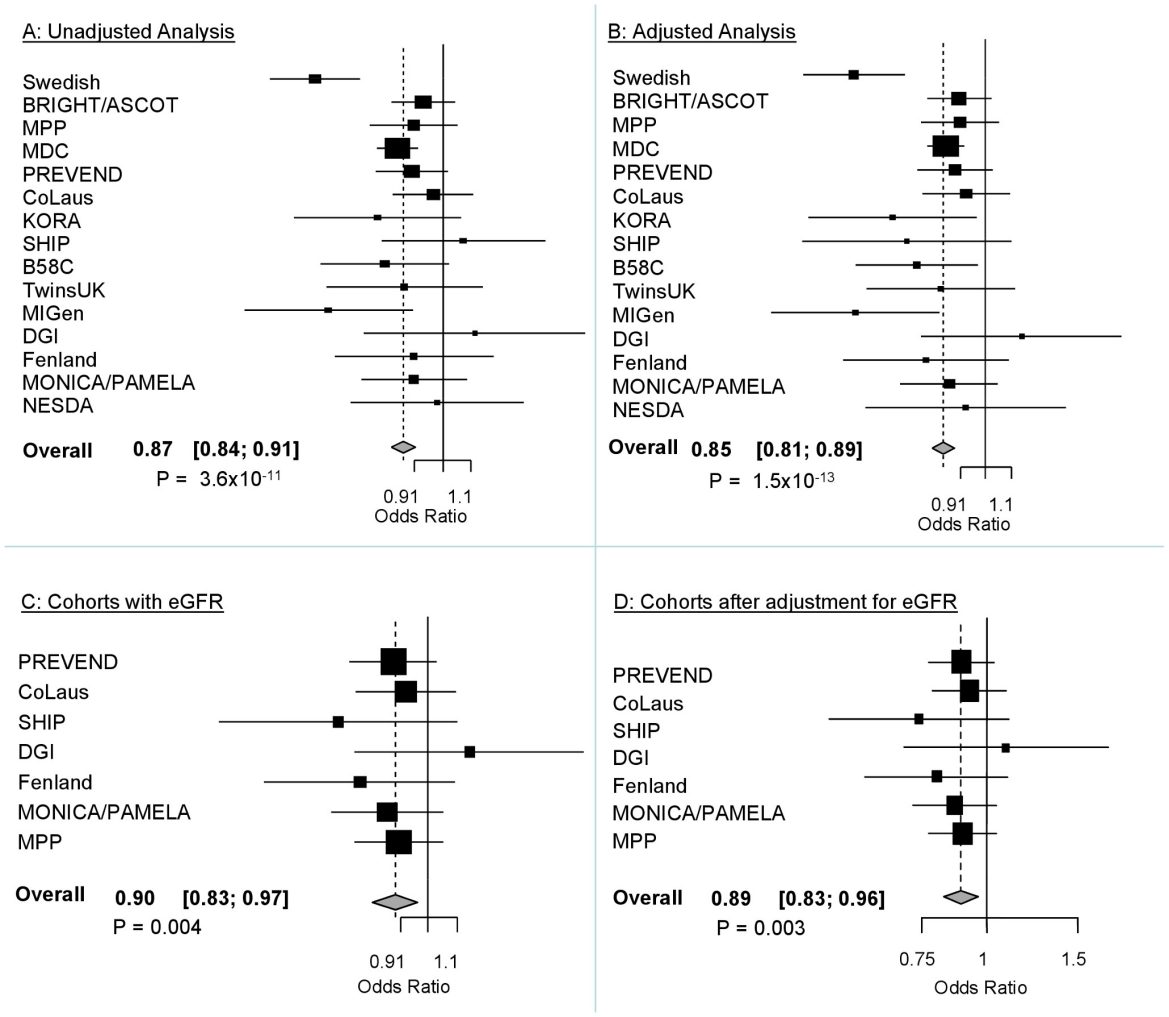
Forest Plots of association with rs13333226 and hypertension (adjustment for population stratification was applied using principal components as appropriate for each cohort). A: Forest plot of association analysis unadjusted for any covariates −21,466 cases and 18,240 controls. B: Forest plot of association analysis adjusted age, age^2^, sex and BMI −21,466 cases and 18,240 controls. C: Forest plot of association analysis in the cohorts where eGFR was available and adjusted for age, age^2^, sex, BMI −7427 controls and 5739 cases. D: Forest plot of association analysis in the cohorts where eGFR was available and adjusted for age, age^2^, sex, BMI and eGFR −7427 controls and 5739 cases.

The demographic characteristics of the discovery and validation cohorts are presented in Table 1 and Table S1 respectively. The results of the GWAS in the discovery sample are presented in Figure 1. The observed versus expected p-value distributions (quantile-quantile plots) are shown in Figure 2. The top hit was rs13333226 with the minor G allele associated with a lower risk of hypertension (OR [95%CI]: 0.6 [0.5–0.73]; p = 1.14×10^−7^; Figure 3) and we selected this for validation in two stages (Figure S1, Table 2 and Table 3). In the first stage we genotyped rs13333226 in the MONICA/PAMELA population samples (in which we also genotyped an additional top 88 SNPs – Table S2) and in the larger MDC and MPP validation case-control populations. For the stage 1 validation, we had 9,827 cases and 8,694 controls and the combined analysis showed the minor G allele to be associated with a lower risk of hypertension (0.87 [0.82–0.92]; p = 3.6×10^−6^) after adjustment for age, age^2^ and BMI. Combined analysis of the 89 SNPs genotyped in the MONICA/PAMELA with the discovery cohort showed rs13333226 (p = 3.86×10^−7^) and rs4293393 (p = 3.30×10^−7^, r^2^ = 0.996) were the top SNPs. In stage 2 analysis which included 10,018 cases and 7,847 controls, the results were similar with the G allele associated with a lower risk of hypertension (0.86 [0.81–0.92]; p = 1.0×10^−5^). Combining stage 1 and stage 2 cohorts increased the strength of association (0.86 [0.83–0.90]; p = 1.61×10^−10^). There was no evidence of heterogeneity across the stage 1 or stage 2 samples or the combined stage 1 and 2 samples as tested by the Q statistic (p>0.05). Merging stages 1 and 2 with the discovery samples yielded the strongest association signal for rs13333226 (0.85 [0.81–0.89]; p = 1.5×10^−13^) with some evidence of heterogeneity (Q statistic p value = 0.04) introduced by the discovery cohort (Table 2, Figure 4A and 4B, Figure S2). This is probably due to the fact that the discovery cohort was ascertained using more extreme criteria than the replication cohorts. In the 13,446 individuals with eGFR measurements available, the strength of association of rs13333226 with hypertension was identical after correcting for eGFR and the effect sizes remained unchanged (unadjusted for eGFR: OR [95%CI]  = 0.90[0.83;0.96], p = 0.004; after eGFR adjustment: OR [95%CI] = 0.89[0.83;0.96], p = 0.003) and there was no evidence of heterogeneity across the study samples (Table 3, Figure 4C and 4D).

**Table 1:** Demographic characteristics of the discovery case control population.

	Controls(n = 1699)	Cases(n = 1621)	p
Age at enrolment, years	57.4 (5.9)	55.4 (7.1)	<0.001
BMI, kg/m^2^	24.2 (3.5)	27.1 (7.8)	<0.001
SBP, mmHg	115.8 (6.8)	175.8 (22.5)	<0.001
DBP, mmHg	73.7 (5.7)	104.7 (11.8)	<0.001

**Table 2:** Results from the meta-analysis of rs13333226 and HTN in discovery sample and after validation.

Study	origin	cases	controls	maf	Unadjusted Analysis		Adjusted for age, age^2^, sex BMI		Q (Unadj/Adj)
					OR [95%-CI]	p	OR [95%-CI]	p	
Swedish BP Extremes (Discovery)	Swedish	1621	1699	0.17	0.65 [0.56–0.76]	1.10×10^−07^	0.6 [0.5–0.73]	3.3×10^−07^	
Stage 1									
MONICA/PAMELA	Italian	894	746	0.19	0.91 [0.76–1.08]	0.282	0.87 [0.72–1.05]	0.145	
MPP	Swedish	1956	1057	0.18	0.91 [0.78–1.05]	0.193	0.91 [0.78–1.05]	0.186	
MDC	Swedish	6977	6891	0.18	0.86 [0.80–0.92]	0.001	0.86 [0.80–0.92]	3.0×10^−05^	
Stage 1 Analysis		9827	8694	0.183	0.87 [0.82–0.93]	6.7×10^−6^	0.87 [0.82–0.92]	3.6×10^−6^	0.73/0.81
Stage 1 + Discovery		21275	19087	0.18	0.84 [0.79–0.89]	4.4×10^−10^	0.84 [0.79–0.89]	2.5×10^−9^	0.01/0.01
Stage 2									
BRIGHT/ASCOT	British/Irish	3069	1787	0.18	0.94 [0.84–1.04]	0.229	0.9 [0.80–1.02]	0.103	
PREVEND	Dutch	2411	1613	0.18	0.9 [0.80–1.02]	0.091	0.89 [0.77–1.03]	0.113	
CoLaus	Swiss	1300	1375	0.19	0.97 [0.84–1.11]	0.634	0.93 [0.79–1.1]	0.375	
KORA	German	457	300	0.16	0.8 [0.61–1.06]	0.128	0.7 [0.51–0.97]	0.03	
SHIP	German	656	240	0.18	1.07 [0.81–1.41]	0.627	0.74 [0.50–1.1]	0.137	
58BC	British	514	529	0.19	0.82 [0.66–1.02]	0.077	0.77 [0.61–0.97]	0.026	
TwinsUK	British	245	845	0.19	0.88 [0.68–1.14]	0.332	0.84 [0.63–1.12]	0.236	
MIGen	European Ancestry	316	278	0.21	0.68 [0.51–0.9]	0.004	0.61 [0.44–0.84]	0.002	
DGI	Swedish/Finnish	277	161	0.23	1.11 [0.77–1.62]	0.572	1.15 [0.78–1.68]	0.483	
Fenland	British	264	510	0.19	0.91 [0.69–1.19]	0.478	0.8 [0.58–1.09]	0.158	
NESDA	Dutch	509	209	0.18	0.98 [0.73–1.31]	0.898	0.93 [0.63–1.35]	0.689	
Stage 2 Analysis		10018	7847	0.189	0.91 [0.86–0.96]	0.0019	0.86 [0.81–0.92]	1.0×10^−5^	0.5/0.3
Stage 2 + Discovery		11639	9546	0.188	0.88 [0.83–0.93]	1.2×10^−6^	0.83 [0.78–0.88]	5.4×10^−9^	0.01/0.02
Combined Analysis - Stage 1 + Stage 2		19845	16541	0.188	0.89 [0.86–0.93]	7.36×10^−08^	0.86 [0.83–0.90]	1.61×10^−10^	0.52/0.51
Combined Analysis - Discovery + Stage 1 + Stage 2		21466	18240	0.187	0.87 [0.84– 0.91]	3.60×10^−11^	0.85 [0.81– 0.89]	1.5×10^−13^	0.02/0.04

Q(unadj/adj)  = P value of the meta-analysis Q test for heterogeneity for the unadjusted and adjusted meta-analysis respectively.

**Table 3:** Results from the meta-analysis of rs13333226 and HTN before and after adjustment for eGFR.

					Adjusted for age, age^2^, sex, BMI		Adjusted for age, age^2^, sex, BMI, eGFR	
	controls	cases	eGFR mean	eGFR SD	OR [95%-CI]	p	OR [95%-CI]	p
PREVEND	2404	1606	80.36	14.39	0.9 [0.77;1.03]	0.113	0.89 [0.77;1.03]	0.135
CoLaus	1375	1298	83.28	16.35	0.93 [0.79;1.1]	0.375	0.93 [0.79;1.1]	0.377
SHIP	240	656	87.62	19.78	0.74 [0.5;1.11]	0.137	0.74 [0.5;1.1]	0.144
DGI	120	141	72.69	11.67	1.09 [0.69;1.72]	0.482	1.094 [0.78;1.68]	0.698
Fenland	508	262	98.92	52.96	0.8 [0.58;1.1]	0.158	0.80 [0.58;1.09]	0.174
MONICA/PAMELA	824	719	84.3	16.59	0.87 [0.72;1.05]	0.145	0.89 [0.74;1.09]	0.278
MPP	1956	1057	88.2	15.1	0.91 [0.78–1.05]	0.186	0.90 [0.78;1.05]	0.179
Combined Analysis	7427	5739			0.899 [0.83; 0.97]	0.0036	0.893 [0.83; 0.96]	0.003

P value of the meta-analysis Q test for heterogeneity = 0.87.

### Association with SBP and DBP

We examined association of rs13333226 with continuous blood pressure measurements in the entire Global BPgen, MPP and MDC cohorts (n = 79,133). Each copy of the G allele of rs13333226 is associated with 0.49 mmHg lower SBP (p = 2.6×10^−5^) and 0.30 mmHg lower DBP ( p = 1.5×10^−5^). The direction of continuous trait effect is consistent with the odds of hypertension.

### Clinical functional studies

The SNP rs13333226 is in close proximity to the uromodulin transcription start site at −1617 base pairs (Figure 3). We studied the association between rs13333226 genotypes and different phenotypes including urinary uromodulin, in 256 hypertensive individuals from the BRIGHT cohort and 110 participants from the population-based HERCULES study. Univariate analyses showed that the G allele was associated with lower excretion of uromodulin in both the BRIGHT and HERCULES studies (Table 4 and Table 5). Each copy of the G allele was associated with 0.2 mg/mmol lower urinary uromodulin corrected for urine creatinine in BRIGHT study (p = 0.007). Each copy of the G allele was also associated with 4.6 ml/min/1.73 m^2^ higher eGFR (p = 0.005) in the BRIGHT cohort. In HERCULES, however, a higher creatinine clearance in GG individuals did not attain statistical significance. In both studies the association of rs13333226 with urinary uromodulin levels persisted on multiple regression analysis adjusting for sex, urine sodium and eGFR (p<0.001).

**Table 4:** Univariate association analysis of rs13333226 in 256 hypertensive patients from the BRIGHT study.

	AA (n = 141)	AG (n = 93)	GG (n = 22)	p-value
Male:Female	0.7	0.8	0.6	0.763
Age (years)	64.7(8.4)	63.9(7.8)	59.5(9.5)	**0.036**
Body mass index (Kg/m^2^)	26.8(4.6)	26.8(5.4)	27.2(3.9)	0.927
Body surface area (m^2^)	1.8 (0.2)	1.9 (0.2)	1.8 (0.2)	0.494
SBP (mm Hg)	156(19.5)	151.5(18.9)	153.3(14.5)	0.205
DBP (mm Hg)	93.1(10)	90.9(10.7)	93.3(10.3)	0.266
Sodium (mmol/L	138.6(3.1)	138.9(3)	137.8(2.9)	0.341
Potassium (mmol/L)	4.4(0.9)	4.2(0.8)	4.4(1)	0.429
Urea (µmol/L)	6.3(1.6)	5.7(1.6)	6(1.6)	**0.025**
Creatinine (µmol/L)	92.2(21.7)	88.4(18.7)	82.9(20)	0.096
Urate (µmol/L)	0.3 (0.1)	0.3 (0.1)	0.3 (0.1)	0.726
eGFR (ml/min/1,73 m^2^)	67.6(16.2)	70.3(12.3)	79.5(15.2)	**0.005**
Creatinine Clearance (ml/min)	70.6 (20.3)	76.2 (20)	86.6 (26.6)	**0.004**
Urine Sodium (mmol/24 h)	139.1(61.9)	158.9(70.6)	142.4(58.3)	0.073
Urine Potassium (mmol/24 h)	66.4(24.1)	78.8(54)	69.2(18.8)	**0.050**
Creatinine excretion (mmol/24 h)	10.2(3.6)	10.8(4.6)	10.7(3.1)	0.520
Uromodulin (mg/L)	5.3(5.3)	5.2(5.5)	3.2(3.4)	0.234
Fractional Excretion Sodium (%)	0.92 (0.37)	0.95 (0.36)	0.73 (0.19)	**0.032**

**Table 5:** Univariate association analysis of rs13333226 in 110 participants from the HERCULES Study.

	AA (n = 52)	AG (n = 46)	GG (n = 12)	p-value
Male:Female (n)	28/24	18/28	7/5	0.258
Age (years)	58 (49–67)	56 (49–66)	59 (49–66)	0.889
Body mass index (Kg/m^2^)	26.1 (23.6–29.3)	24.4 (21.8–29)	24.7 (24–28)	0.175
Body surface area (m^2^)	1.84 (1.72–1.98)	1.76 (1.62–1.92)	1.87 (1.77 – 2.00)	**0.045**
24 h SBP (mm Hg)	115.4 (107.7–123.0)	113.2 (105.9–124.8)	118.4 (111.4–130.7)	0.555
24 h DBP (mm Hg)	76.3 (69.8–81.1)	77.1 (71.5–85.2)	77.7 (71.1–87.7)	0.547
Hypertension* (%)	33	30	25	0.846
Fasting plasma				
Sodium (mmol/L)	139.7 (138.1–141.8)	139.9 (138.2–141.5)	140.3 (138.4–142.7)	0.708
Potassium (mmol/L)	4.0 (3.8–4.3)	4.0 (3.7–4.1)	3.8 (3.6 – 4.0)	**0.041**
Urea (µmol/L)	5.3 (4.4–6.1)	4.8 (4.4–6)	4.4 (4.1 – 5.0)	0.141
Creatinine (µmol/L)	82 (73.5 – 91.5)	81 (73–88)	76.5 (72.5–80.5)	0.225
Urate (µmol/L)	318 (290–378)	321 (262–373)	294 (268–318)	0.163
24 h urine				
Uromodulin (mg/L)	30.8 (15.6–51.7)	24.5 (14.2–42.5)	14 (10.6–16.5)	**0.005**
Uromodulin (mg/24 h)	53 (25–76)	40 (28–68)	17 (14–33)	**0.005**
Urine volume (mL)	1725 (1200–2375)	1665 (1150–2100)	1773 (1125–2300)	0.864
Creatinine clearance (mL/min)	98.9 (70.5–123)	93.7 (75.4–123.3)	109.4 (86.2–125.1)	0.607
Creatinine excretion (mmol/kg/24 h)	0.15 (0.12–0.19)	0.16 (0.13–0.19)	0.16 (0.12–0.19)	0.745
Urine Sodium (mmol/24 h)	138 (86–176)	134 (92–175)	109 (84–161)	0.785
Urine Potassium (mmol/24 h)	59 (37–84)	61 (45–74)	48 (39–80)	0.865
Fractional Excretion Sodium (%)	1.2 (0.6–1.8)	1.2 (0.8–1.7)	0.7 (0.6–1.7)	0.696
Fractional Excretion of Lithium (%)	0.10 (0.07–0.17)	0.15 (0.09–0.21)	0.11 (0.06–0.15)	**0.031**

Data are median (interquartile range).

*Hypertension defined based on 24 hour ambulatory blood pressure (>135/85 or on antihypertensive treatment).

In BRIGHT, GG carriers were found to have a significantly lower fractional excretion of sodium (p = 0.032). In the smaller HERCULES sample this also occurred, though short of statistical significance. However, in HERCULES urinary uromodulin was positively associated with urinary sodium excretion (p = 0.025) and fractional excretion of endogenous lithium (r^2^ = 0.19, p = 0.045). Overall, BRIGHT and HERCULES data suggest that low urinary uromodulin is associated with higher sodium reabsorption, and that this occurs at the proximal tubular level.

In the small GRECO cohort, urinary uromodulin concentration (p = 0.004) and 24 hour uromodulin excretion (p = 0.002; Wilcoxon′s signed ranks test) were found to be significantly increased after a high sodium intake (Table 6). The G allele was associated with lower uromodulin excretion only on low sodium diet (p = 0.002).

**Table 6:** Univariate association analysis of urinary uromodulin in relation to rs13333226 polymorphism and response to high and low sodium intake (GRECO Study).

	AA (n = 40)	AG and GG (n = 24)	p-value
Male:Female (n)	40/0	24/0	1.0
Age (years)	26 (8)	23 (6)	0.105
Body mass index (Kg/m^2^)	23.4 (2.7)	23.4 (2.1)	1.0
Body surface area (m^2^)	2.05 (0.14)	2.03 (0.15)	0.590
SBP LS (mm Hg)	120 (10)	121 (10)	0.670
DBP LS (mm Hg)	68 (9)	70 (6)	0.453
SBP HS (mm Hg)	123 (10)	124 (10)	0.805
DPB HS (mm Hg)	69 (8)	70 (7)	0.661
GFR LS (mL/min/1.73 m^2^)	109 (13)	103 (14)	0.127
GFR HS (mL/min/1.73 m^2^)	114 (14)	116 (15)	0.719
ERPF LS (mL/min/1.73 m^2^)	472 (74)	449 (68)	0.209
ERPF HS (mL/min/1.73 m^2^)	502 (90)	489 (68)	0.529
ECV LS (L/1.73 m^2^)	16.5 (1.9)	16.3 (1.6)	0.657
ECV HS (L/1.73 m^2^)	17.2 (1.7)	18.0 (1.9)	0.093
Fractional Excretion Sodium LS (%)	0.19 (0.18)	0.22 (0.25)	0.342
Fractional Excretion Sodium HS (%)	0.99 (0.35)	0.82 (0.31)	**0.001**
Plasma Renin Activity LS (nmol/L/h)	6.3 (3.7)	6.6 (3.1)	0.723
Plasma Renin Activity HS (nmol/L/h)	2.5 (1.5)	2.0 (0.9)	0.155
Uromodulin LS median (IQR) (mg/L)	10.3 (6.9–15.6)	9.0 (6.3–14.2)	**0.002**
Uromodulin HS median (IQR) (mg/L)	11.9 (7.5–27.9)	12.2 (7.2–21.3)	0.513

LS: Low sodium diet, HS: High sodium diet; Data are presented as mean (SD). P-value comparing AA versus AG+GG.

### Cardiovascular outcomes and rs13333226

Finally, we evaluated the clinical significance of our findings by testing whether the low BP associated allele may protect against development of cardiovascular events during long-term follow-up at the population level. Among 26,654 subjects from the entire population based MDC study [11] who were free from prior cardiovascular events at baseline, 2,750 individuals developed cardiovascular events (CVD) during 12 years of follow-up. We found each copy of the G allele to be associated with a 7.7% reduction in risk of CVD events after adjusting for age, sex, BMI and smoking status (H.R.  = 0.923, 95% CI 0.860–0.991; p = 0.027). When SBP (H.R.  = 0.936, 95% CI 0.872–1.005; p = 0.067) or SBP and DBP (H.R.  = 0.937, 95% CI 0.873–1.005; p = 0.069) were added to the Cox regression model, the directionality and risk remained almost identical.

## Discussion

We have identified and validated a SNP upstream of the uromodulin (*UMOD*) gene whose minor allele is associated with a lower risk of hypertension. The associated SNP (rs13333226) is in close proximity to the uromodulin transcription start site at −1617 base pairs. There is only one previous candidate gene study of *UMOD* and hypertension. This study tested rs6497476, located in the 5′ region of the *UMOD* gene (−744 bp from *UMOD* transcriptional start point) and showed the minor allele with a lower risk of hypertension in a Japanese population, but it did not reach statistical significance [12]. This SNP is correlated with rs13333226 in the Japanese HapMap population (r^2^ = 0.91) and shows the same directionality of effect. A recent genome scan for chronic kidney disease (CKD) [13] has found the minor T allele at rs12917707, −3653 bp upstream from the *UMOD* transcription start site to be associated with a 20% reduction in risk of CKD. This association was consistent after adjusting for major CKD risk factors including SBP and hypertension. This SNP -rs12917707 is perfectly correlated (r^2^ = 1 in HapMap CEU) with rs13333226. Our data show the minor allele of rs13333226 is associated with increased eGFR (beta = 3.6, p = 0.012), but adjustment for eGFR in our meta-analyses did not alter its association with lower risk for hypertension. This suggests that the UMOD locus is independently associated with hypertension. We also show an association of rs13333226 with long term cardiovascular outcomes with a relatively small attenuation of the relationship after SBP/DBP adjustment. This suggests UMOD may have an influence on cardiovascular disease at least partly independent of BP. However, our conditional analyses are limited by the fact that single point measures of BP and eGFR may not truly represent the lifetime effect of the genetic variant on these traits. Therefore, we cannot exclude that rs13333226 may exert its effects on hypertension and cardiovascular disease, at least partly through its effects on renal function and blood pressure, respectively.

The *UMOD* gene encodes the Tamm Horsfall protein (THP)/uromodulin, a glycosylphosphatidylinositol (GPI) anchored glycoprotein. It is the most abundant tubular protein in the urine, which is expressed primarily in the thick ascending limb of the loop of Henle (TAL) with negligible expression elsewhere [14], [15]. We show in the BRIGHT, HERCULES and GRECO (low sodium diet) that the minor allele of rs13333226 (associated with a lower risk of hypertension) is consistently associated with lower urinary uromodulin excretion. This effect was lost when GRECO subjects were given a high sodium diet. We also show in BRIGHT and HERCULES that the G allele and lower urinary uromodulin are associated with lower fractional excretion of sodium and lower fractional excretion of endogenous lithium, indicating increased sodium reabsorption at the proximal tubular level. While the association of lower blood pressure and increased sodium reabsorption may appear counterintuitive, an increased sodium reabsorption by the proximal tubule may simply be the consequence of an increased sodium load because of increased GFR, or a compensatory reaction to a primary decrease in distal reabsorption. In absence of information on sodium intake in individuals in BRIGHT and HERCULES, we cannot exclude that the lower fractional sodium excretion in carriers of the G allele simply reflects a low dietary sodium intake. The exclusive expression of uromodulin in TAL, where physiologically crucial mechanisms of sodium handling are located, suggests that alterations of some of these mechanisms in G allele carriers may underlie their lower risk of hypertension. However, functional studies are needed to clarify the renal mechanisms by which the *UMOD* gene may affect hypertension and renal sodium handling.

In the context of our findings it is of interest to note that *UMOD* mutations (in exons 4 and 5) are implicated in monogenic syndromes such as familial juvenile hyperuricemic nephropathy, autosomal-dominant medullary cystic kidney disease [MCKD2] and glomerulocystic kidney disease (GCKD) (MIM603860, MIM162000, MIM609886) [16]–[18]. In previous small studies, urinary uromodulin levels were found to be decreased in older subjects and in subjects with renal impairment [19], [20]. In renal disease patients, uromodulin excretion was reduced in proportion to the extent of renal damage, and was a marker of distal tubular sodium reabsorption, but in these studies, the effects of BP on uromodulin were inconsistent [21], [22]. The TAL, where *UMOD* is selectively expressed is also the site where mutations of tubular transporters have resulted in rare Mendelian high or low BP syndromes [23]. Furthermore, recent data from Lifton's group demonstrated that heterozygous mutations in *SLC12A3* (encoding the thiazide-sensitive Na-Cl cotransporter), *SLC12A1* (encoding the Na-K-Cl cotransporter NKCC2), and *KCNJ1* (encoding the K+ channel ROMK) discovered in the general population have been associated with lower BP and a 60% reduction in the development of hypertension [24].

Our strategy of using extremes of BP distribution has led to the discovery of a gene variant that could not be discovered when a less stringent case-control definition was used [10]. For example, in stage 1 Global BPgen samples (n = 34,433), the p values for association of rs13333226 with SBP and DBP were 0.0077 and 0.0099 respectively indicating that rs13333226 would not have been selected for validation as the p-value threshold for follow-up genotyping in that study was p<10^−5^. Also, in Global BPgen study when the top 8 SNPs that attained genome wide significance for continuous BP were tested for association with hypertension, four of the eight SNPs had 0.01<p≤0.10 with odds of hypertension in directions consistent with the continuous trait effect. As effect size of the risk allele of rs13333226 is comparable to the effect sizes of the previous robust association signals for blood pressure[8], [9], we think that using an extreme case-control strategy successfully enabled the discovery of a locus that previous GWAS meta-analysis failed to detect possibly due to the cost imposed by multiple testing correction.

The main limitation of our study is that the functional studies were performed on three different populations – hypertensive, population-based and dietary sodium intervention samples. The renal and blood pressure measurements were measured at single time-points and are not entirely representative of genotype-phenotype effects which occur over prolonged time periods. On the other hand, definitions of the extreme hypertension and extreme normotension in the discovery cohort are based on very robust data. Subjects with extreme hypertension were chosen from an intervention trial in which blood pressure was measured after a wash-out period during which all antihypertensive therapy was discontinued before randomization, whereas normotensive controls were chosen from a population followed up for 10 years and who remained free of cardiovascular disease and antihypertensive treatment throughout this period. Therefore, we think that the newly discovered UMOD locus for hypertension has the potential to give unique insights into the mechanisms of high blood pressure, and identify novel drugable targets.

## Methods

### Ethical considerations

All studies were approved by institutional ethics review committees at the relevant organizations. All participants provided informed written consent.

### Discovery cohort

To identify novel susceptibility loci for hypertension, we used an extreme case-control design. Hypertensive cases had to have at least two consecutive BP measurements of ≥160 mmHg systolic and ≥100 mmHg diastolic, with the diagnosis made before age 63 years. We identified 2,000 cases in the Nordic Diltiazem study (NORDIL) [25]. These hypertensive subjects represent approximately the top 2% of the BP distribution in the Swedish population. Two thousand control subjects were drawn from the Malmö Diet and Cancer study (MDC, n = 27,000) [26] who had a SBP≤ 120 mmHg and DBP≤ 80 mmHg. Controls had to be at least 50 years of age and free from cardiovascular events (coronary events and stroke) during 10 years of follow up [27] and not on any antihypertensive medication. The controls derived from the MDC population represented the lower 9.2% of the BP distribution and with the selection for low cardiovascular risk, can be considered as hyper-controls. In both NORDIL and MDC, BP was measured in the recumbent position after 5–10 minutes rest using a manual sphygmomanometer. Rigorously phenotyped samples minimize case/control misclassification, and the potential advantage of an extreme case/control design is greater power to detect variants associated with BP and hypertension, for a given total sample size and total genotyping cost.

### Validation cohorts

For the validation we used phenotypic definitions (extreme SBP/DBP thresholds) to closely match our discovery samples. The BP measurements in all the cohorts were based on the average of at least 2 measurements obtained when the subject was seated and after rest for at least 5 minutes. The BP criteria were slightly modified as most validation cohorts were general population cohorts. Cases: Individuals less than 60 years of age with SBP ≥140 mmHg or DBP ≥90 mmHg or current treatment with antihypertensive or BP lowering medication commenced before age 60 years. Controls: Individuals with SBP ≤120 mmHg and DBP ≤80 mmHg, at least 50 years of age, and free from any BP lowering medication. If age ≤50 years, then the criteria were slightly modified to SBP ≤115 mmHg and DBP ≤80 mmHg and free from BP lowering medications. The validation cohorts were the MONItoring trends and determinants of CArdiovascular diseases (MONICA)/Pressioni Arteriose Monitorate E Loro Associazioni (PAMELA) studies (894 cases/746 controls) from Northern Italy [6], [28], 1956 cases/1057 controls from the 2002–2006 follow-up exam of the Malmö Preventive Project (MPP) [29] and 6977 cases/6891 controls from the Malmö Diet and Cancer study [11] (MDC; non-overlapping with discovery samples), 509 cases/209 controls from The Netherlands Study of Depression and Anxiety study (NESDA) [30] and ten cohorts from a collaboration with the Global BPgen consortium [9]. Analyses reported here are distinct from those previously published [9], because they use phenotypic definitions to match our discovery samples. The combined sample size of the discovery and validation cohorts is 39,706 individuals (21,466 cases and 18,240 controls).

Estimated glomerular filtration rate (eGFR) was calculated using the Modification of Diet in Renal Disease (MDRD) Study equation [31].

### Clinical functional studies

We studied functional associations of the top SNP in a hypertensive cohort and a population cohort with extensive urine phenotypes and one interventional study of low and high sodium intake with extensive measurements of sodium balance.

The British Genetics of Hypertension (BRIGHT) study [32] is a hypertension case-control study. Case inclusion criterion was a diagnosis of hypertension (>150/100 mmHg) prior to 50 years of age. Exclusion criteria included BMI>35, diabetes, secondary hypertension or co-existing illness. 24-hour urine collection was available for all the cases with measurements of urinary sodium, potassium, creatinine and microalbuminuria. We measured urinary uromodulin in 256 hypertensive subjects.

Groningen Renal Hemodynamic Cohort Study Group (GRECO): The GRECO protocol comprises integrated measurement of renal hemodynamics and extracellular volume as applied in an ongoing series of studies in healthy subjects [33], [34]. For the current analysis 64 healthy adult males were included (mean age = 23 years), who had been studied after two seven-day periods: the first 7 days on a low sodium diet (LS, 50 mmol Na^+^ per day, balance verified by repeated 24 h urine), the second 7 days on a high-sodium diet (HS, 200 mmol Na^+^ per day).

Hypertension Evaluation by Remler and CalciUria LEvel Study (HERCULES) is a substudy of the population-based CoLaus study (www.colaus.ch) from Lausanne Switzerland [35], [36]. A random sample of 411 CoLaus participants, aged 38–78 years, underwent ambulatory BP monitoring and 24 hour urine collection. The phenotypes available include 24-hour urine collection with measurement of creatinine clearance, endogenous lithium clearance, urinary sodium, potassium and uric acid excretion and microalbuminuria. We measured urinary uromodulin in 110 participants of this study.

### Urinary uromodulin measurements

Urinary uromodulin was measured in duplicate in 24 hour urine samples using a commercially available ELISA (MD Biosciences, Zürich, Switzerland) as recommended by the manufacturer. The range of assay is 9.375–150 ng/mL and sensitivity <5.50 ng/mL. The inter-assay coefficient of variation was 11.9%. Urinary uromodulin levels were corrected for urine creatinine before analysis.

### Genotyping and quality control

The genomewide association study (GWAS) samples were genotyped using Illumina 550K Single and Illumina 610 Quad V1 BeadChip (Illumina, Inc., San Diego, CA, USA). We included 551,629 SNPs common to both the Single and Quad chips, for analysis. SNPs with a minor allele frequency (MAF) <1% or in significant Hardy-Weinberg disequilibrium (P<1×10^−7^) in pooled samples were removed leaving 521,220 SNPs for analysis. We assessed population structure within the data using principal components analysis as implemented in EIGENSTRAT [37] to infer continuous axes of genetic variation. After data quality control for unspecified sex (5 subjects removed), relatedness/duplicates (68 individuals removed), multidimensional scaling plot outliers (33 individuals removed), genetic outliers - which are defined as individuals whose ancestry is at least 6 s.d. from the mean on one of the top ten axes of variation on principal component analysis (388 individuals removed) and genotyping success of <95% (92 individuals removed), genotype information from 1,621 cases and 1,699 controls (1,510 males and 1,810 females) was available for analysis. Untyped SNPs were imputed using IMPUTE v1 [38] with data from the August 2009 release of CEU phased haplotypes from Pilot 1 of the 1000 Genomes Project NCBI Build 36 (dbSNP b126) as the reference panel (from https://mathgen.stats.ox.ac.uk/impute/impute_v1.html). The probability threshold used for calling an imputed genotype was 0.9. Association analysis was performed using SNPTEST [38] taking into account uncertainty in imputation.

### Statistical analysis

In the GWAS samples, we tested each SNP for association using an additive genetic model and logistic regression with adjustment for significant ancestry principal components [37] to correct for population stratification. There was still a slight overall inflation of test statistics, with a genomic control inflation factor (λ) of 1.07 (Figure 2). All results are presented after application of genomic control to correct for this residual inflation [39]. Additionally two logistic regression analyses were performed, with adjustment for age, age^2^, sex and BMI and with adjustment for age, age^2^, sex, BMI and eGFR. Multiple linear regression was used to test association between genotype and urinary uromodulin levels, functional parameters like GFR, extracellular volume etc. with relevant covariates. In the GRECO study, as the numbers of GG genotypes were small, AG and GG were combined for analysis. Non-normally distributed traits were tested using the non-parametric Kruskal Wallis test.

### Validation analysis

In validation samples, SNPs were tested for association using logistic regression, with adjustment for ancestry principal components where available to correct for population stratification. Meta-analysis of the combined discovery and validation results was conducted using an inverse-variance weighted (fixed-effects) meta-analysis.

In the meta-analysis, a genomewide significance threshold of 5×10^–8^ corresponding to a P value of 0.05 with a Bonferroni correction for 1 million independent tests was considered *a priori* as genomewide significant [40].

### Continuous BP trait modeling

The associations between the validated SNP and SBP and DBP were analysed separately in the Stage 1 samples of the Global BPgen consortium (n = 34,433) and in the overall MDC (n = 27,000) and MPP (n = 17,700) cohorts [9],[26],[29]. The results were combined using fixed-effect inverse variance weighted meta-analysis. Continuous SBP and DBP were adjusted for age, age^2^, body mass index and any study-specific geographic covariates in sex-specific linear regression models. In individuals taking antihypertensive therapies, blood pressure was imputed by adding 15 mm Hg and 10 mm Hg for SBP and DBP, respectively [9], [41].

### Survival analysis

We performed multivariable Cox proportional hazards models to examine the association between biomarkers and incident events. (myocardial infarction, stroke, coronary death). Two models, one adjusted for age, sex, BMI , SBP and smoking status and another adjusting for age, sex, BMI , SBP, DBP and smoking status were analysed. We confirmed that the proportionality of hazards assumption was met. The results are presented as hazard ratios and 95% confidence intervals per copy of the G allele. Survival analysis was performed using SPSS version 13.0 for Windows (SPSS Inc).

## Supporting Information

Figure S1 Study design showing the discovery and two validation stages with the SNPs genotyped in each cohort along with sample sizes.
(0.32 MB TIF)

Figure S2 A: Funnel Plot of all cohorts including discovery samples. Test of heterogeneity: p = 0.02. B: Funnel Plot of all cohorts excluding discovery samples. Test of heterogeneity: p = 0.52.
(0.11 MB TIF)

Table S1 Summary demographics of the validation cohorts.
(0.04 MB DOC)

Table S2 Replication analysis in the Italian MONICA/PAMELA population. Results presented are the discovery, replication and combined analysis using inverse variance fixed effect meta-analysis.
(0.15 MB DOC)

Text S1 Acknowledgments.
(0.06 MB DOC)
