# Supplementary figures and images for: Genome-Wide Association Study of Blood Pressure Extremes Identifies Variant near UMOD Associated with Hypertension
Source: PLoS Genet. 2010 Oct 28;6(10):e1001177. doi: 10.1371/journal.pgen.1001177 (PMC2965757; doi:10.1371/journal.pgen.1001177)

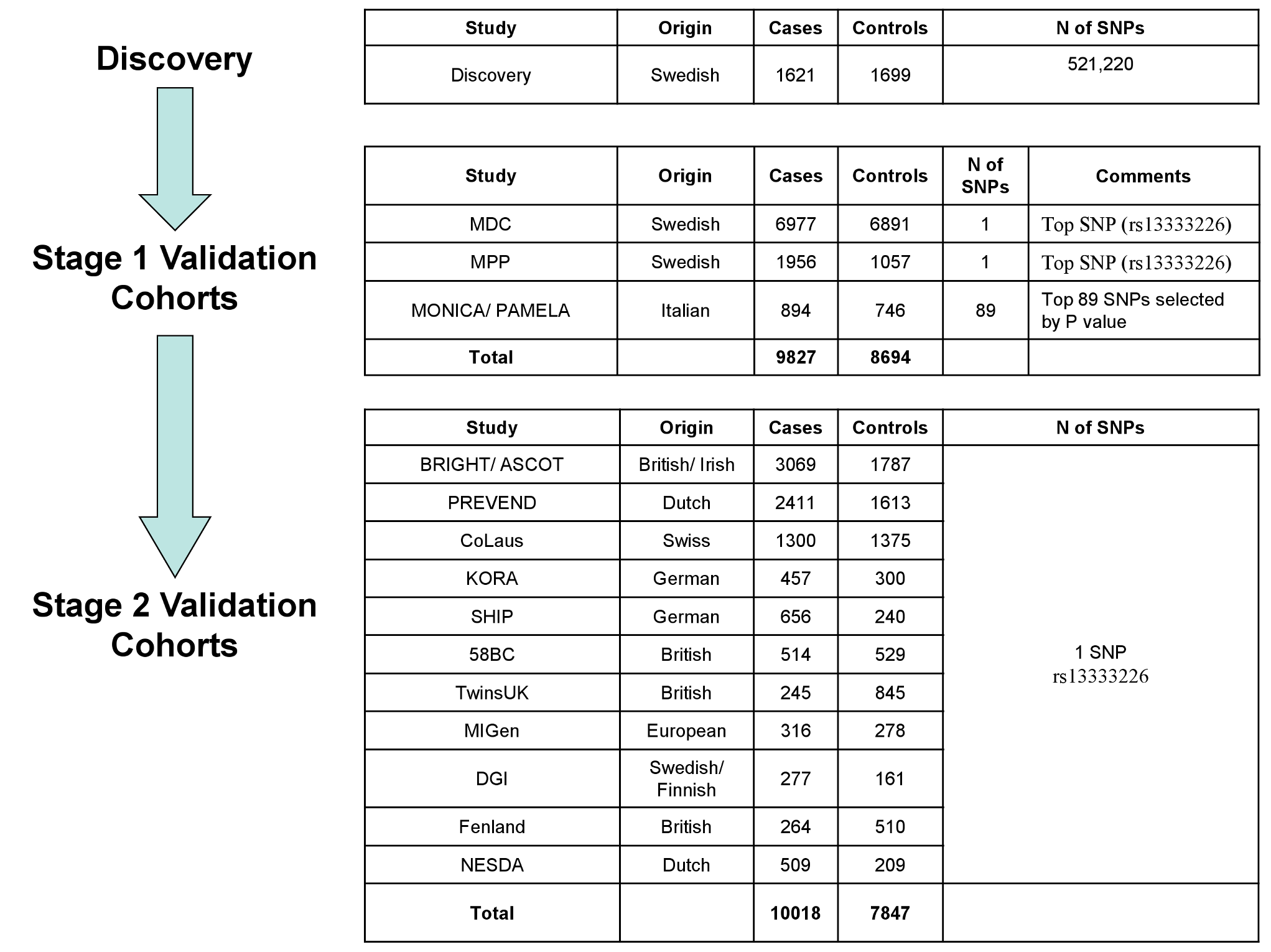

Supplement: Figure S1 — Study design showing the discovery and two validation stages with the SNPs genotyped in each cohort along with sample sizes. (0.32 MB TIF) [file pgen.1001177.s001.tif]

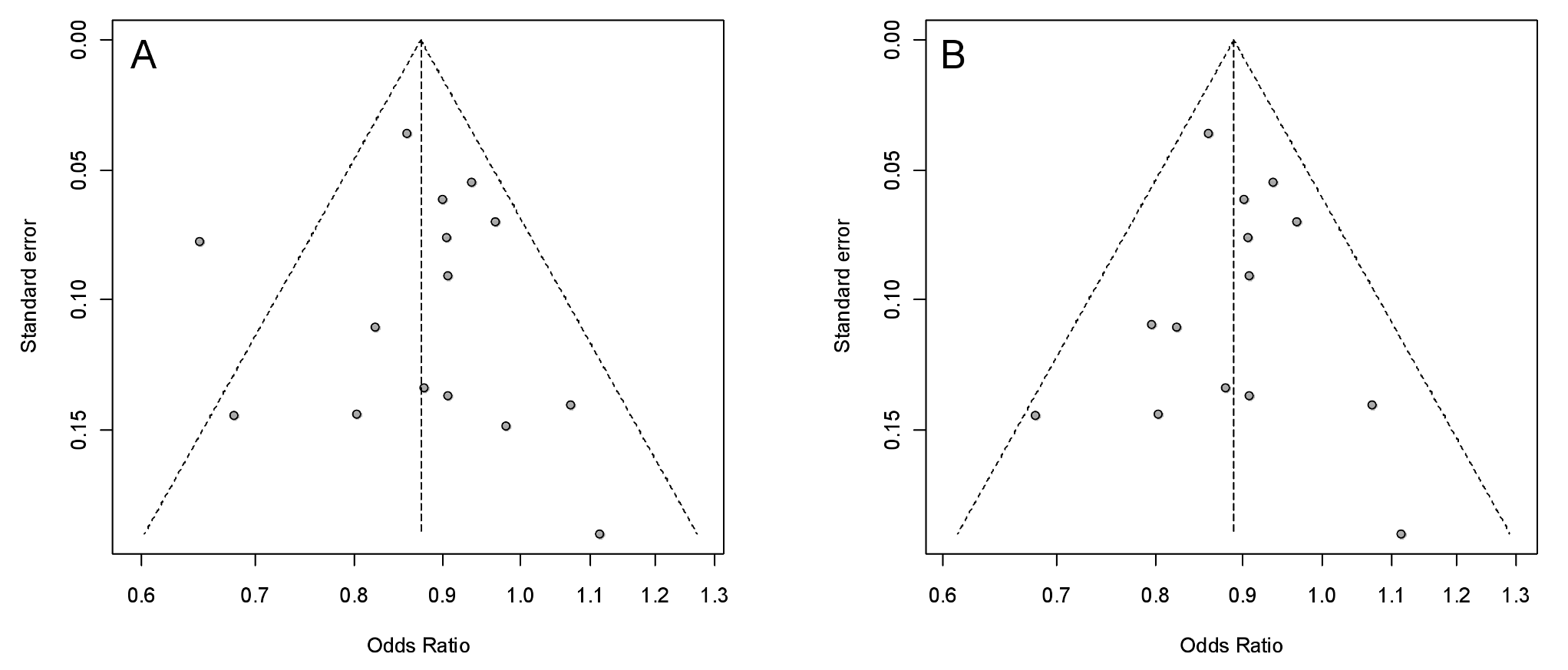

Supplement: Figure S2 — A: Funnel Plot of all cohorts including discovery samples. Test of heterogeneity: p = 0.02. B: Funnel Plot of all cohorts excluding discovery samples. Test of heterogeneity: p = 0.52. (0.11 MB TIF) [file pgen.1001177.s002.tif]
