# Supplementary material for: Genome-Wide Association Study of Blood Pressure Extremes Identifies Variant near UMOD Associated with Hypertension
Source: PLoS Genet. 2010 Oct 28;6(10):e1001177. doi: 10.1371/journal.pgen.1001177 (PMC2965757; doi:10.1371/journal.pgen.1001177)
Supplement: Table S1 — Summary demographics of the validation cohorts. (0.04 MB DOC) [file pgen.1001177.s003.doc]

Table S1: Summary demographics of the validation cohorts. Data presented as mean (SD)

| Study | Controls | | | | | Cases | | | | |
| --- | --- | --- | --- | --- | --- | --- | --- | --- | --- | --- |
|  | N | Age, years | BMI, kg/m^2^ | SBP, mmHg | DBP, mmHg | N | Age, years | BMI, kg/m^2^ | SBP, mmHg | DBP, mmHg |
| BRIGHT/ ASCOT | 1787 | 58.7 (8.92) | 25.2 (3.26) | 123.0 (10.47) | 76.3 (7.19) | 3069 | 60.0 (9.77) | 28.1 (4.22) | 165.6 (20.35) | 99.1 (11.92) |
| MPP | 1057 | 65.7 (6.4) | 25.3 (3.4) | 120.4 (6.8) | 72.7 (4.9) | 1956 | 67.4 (6.3) | 28.3 (4.1) | 169.8 (15.6) | 98.5 (7.0) |
| MDC | 6891 | 54.3 (6.7) | 24.3 (3.4) | 119.9 (7.7) | 75.1 (5.1) | 6977 | 60.8 (7.5) | 27.0 (4.3) | 165.4 (13.5) | 97.2 (6.6) |
| PREVEND | 1613 | 44.6 (10.5) | 24.1 (3.4) | 109.1 (6.1) | 65.9 (5.9) | 2411 | 47.6 (7.7) | 27.9 (4.7) | 142.3 (17.2) | 80.9 (9.6) |
| CoLaus | 1375 | 49.1 (9.2) | 23.6 (3.6) | 108.8 (6.7) | 68.8 (6.1) | 1300 | 54.8 (8.8) | 28.2 (4.9) | 141.9 (16.2) | 88.1 (10.8) |
| KORA | 300 | 46.3 (9.2) | 25.1 (3.7) | 109.9 (6.2) | 70.4 (5.6) | 457 | 51.0 (6.6) | 28.7 (4.1) | 147.5 (15.5) | 91.3 (9.6) |
| SHIP | 240 | 62.1 (9.0) | 26.5 (3.9) | 110.7 (7.1) | 70.1 (6.4) | 656 | 48.2 (7.8) | 29.4 (5.2) | 144.5 (15.5) | 91.7 (9.5) |
| 58BC | 529 | 44.9 (0.3) | 25.6 (4.1) | 108.7 (5.0) | 68.3 (5.2) | 514 | 45.0 (0.3) | 29.4 (5.5) | 148.1 (11.9) | 92.7 (8.2) |
| TwinsUK | 845 | 45.7 (11.8) | 24.8 (4.6) | 117.5 (13.3) | 74.8 (8.8) | 245 | 47.2 (12.1) | 25.1 (4.7) | 139.3 (16.0) | 88.3 (11.7) |
| MIGen | 278 | 45.9 (7.0) | 25.0 (4.0) | 107.3 (7.1) | 69.7 (7.0) | 316 | 48.9 (5.9) | 29.0 (5.5) | 141.4 (14.0) | 89.5 (11.3) |
| DGI | 161 | 60.1 (7.4) | 25.5 (3.2) | 113.2 (6.8) | 70.4 (6.7) | 277 | 52.7 (5.7) | 27.6 (3.7) | 145.8 (15.0) | 87.6 (8.9) |
| Fenland | 510 | 44.1 (7.4) | 25.4 (4.6) | 107.2 (6.8) | 66.7 (6.2) | 264 | 48.8 (6.4) | 29.5 (4.9) | 143.7 (14.1) | 88.0 (9.4) |
| MONICA/ PAMELA | 746 | 56.1(5.2 | 25.4(3.8) | 119.6(8.5) | 78.4(7.4) | 894 | 55.8(7.2) | 27.6(4.4) | 156.6(20.1) | 94.3(10.7) |
| NESDA | 209 | 38.6 (11.8) | 22.9 (3.4) | 111 (4.2) | 69.9 (5.2) | 509 | 46.6 (10.9) | 27.1 (5.1) | 149.6 (14.8) | 88.8 (9.5) |
