# Supplementary material for: Genome-Wide Association Study of Blood Pressure Extremes Identifies Variant near UMOD Associated with Hypertension
Source: PLoS Genet. 2010 Oct 28;6(10):e1001177. doi: 10.1371/journal.pgen.1001177 (PMC2965757; doi:10.1371/journal.pgen.1001177)
Supplement: Table S2 — Replication analysis in the Italian MONICA/PAMELA population. Results presented are the discovery, replication and combined analysis using inverse variance fixed effect meta-analysis. (0.15 MB DOC) [file pgen.1001177.s004.doc]

Table S2: Replication Analysis in the Italian MONICA/PAMELA population. Results presented are the discovery, replication (MONICA/PAMELA) and combined analysis using inverse variance fixed effect meta-analysis.

|  | | | | DISCOVERY | | | | MONICA/PAMELA | | | | COMBINED-ANALYSIS | | | |
| --- | --- | --- | --- | --- | --- | --- | --- | --- | --- | --- | --- | --- | --- | --- | --- |
| CHR | SNP | BP | A1 | N | OR | 95%CI | P | N | OR | 95%CI | P | P-FIXED | OR-FIXED | Q | I |
| 1 | rs1399291 | 97349510 | T | 3315 | 1.26 | 1.13-1.41 | 3.12E-05 | 1611 | 1.08 | 0.94-1.25 | 2.71E-01 | 2.55E-05 | 1.26 | 0.45 | 0 |
| 1 | rs10857978 | 1.13E+08 | T | 3319 | 0.76 | 0.67-0.86 | 3.33E-05 | 1616 | 0.99 | 0.83-1.18 | 8.93E-01 | 3.49E-05 | 0.76 | 0.63 | 0 |
| 2 | rs2192615 | 48975598 | G | 3319 | 0.8 | 0.71-0.89 | 7.77E-05 | 1612 | 0.96 | 0.84-1.11 | 5.88E-01 | 8.09E-05 | 0.8 | 0.63 | 0 |
| 2 | rs12611661 | 1.05E+08 | C | 3314 | 0.76 | 0.66-0.86 | 2.61E-05 | 1618 | 1.08 | 0.92-1.26 | 3.41E-01 | 3.33E-05 | 0.76 | 0.26 | 21.95 |
| 2 | rs9636284 | 1.62E+08 | T | 3319 | 0.63 | 0.52-0.78 | 1.48E-05 | 1615 | 1.15 | 0.93-1.43 | 2.08E-01 | 2.61E-05 | 0.64 | 0.14 | 54.82 |
| 2 | rs2084543 | 1.62E+08 | A | 3320 | 0.6 | 0.48-0.74 | 3.36E-06 | 1616 | 1.18 | 0.94-1.49 | 1.54E-01 | 6.47E-06 | 0.61 | 0.12 | 58.89 |
| 2 | rs16846179 | 1.62E+08 | G | 3320 | 0.63 | 0.51-0.78 | 1.49E-05 | 1618 | 1.12 | 0.9-1.39 | 2.99E-01 | 2.14E-05 | 0.64 | 0.24 | 28.52 |
| 3 | rs9853991 | 32441801 | T | 3316 | 0.71 | 0.61-0.82 | 8.98E-06 | 1615 | 1.01 | 0.84-1.22 | 8.87E-01 | 9.53E-06 | 0.71 | 0.6 | 0 |
| 3 | rs3888882 | 32472578 | T | 3320 | 0.73 | 0.63-0.85 | 2.92E-05 | 1614 | 1.00 | 0.84-1.2 | 9.67E-01 | 2.98E-05 | 0.73 | 0.77 | 0 |
| 3 | rs12636240 | 1.15E+08 | G | 3319 | 1.26 | 1.12-1.4 | 6.15E-05 | 1615 | 1.03 | 0.89-1.19 | 7.21E-01 | 6.20E-05 | 1.26 | 0.84 | 0 |
| 3 | rs9881563 | 1.15E+08 | C | 3318 | 1.26 | 1.13-1.41 | 5.16E-05 | 1613 | 1.01 | 0.87-1.17 | 9.34E-01 | 5.00E-05 | 1.26 | 0.95 | 0 |
| 3 | rs9865965 | 1.15E+08 | T | 3317 | 1.26 | 1.13-1.41 | 4.28E-05 | 1615 | 1.01 | 0.87-1.17 | 8.94E-01 | 4.25E-05 | 1.26 | 0.99 | 0 |
| 3 | rs13061150 | 1.15E+08 | A | 3320 | 1.25 | 1.12-1.4 | 6.88E-05 | 1615 | 1.02 | 0.88-1.18 | 7.94E-01 | 6.90E-05 | 1.25 | 0.86 | 0 |
| 3 | rs9828099 | 1.15E+08 | C | 3320 | 1.25 | 1.12-1.4 | 7.92E-05 | 1615 | 1.03 | 0.89-1.19 | 7.06E-01 | 7.59E-05 | 1.25 | 0.79 | 0 |
| 3 | rs3811647 | 1.35E+08 | A | 3320 | 0.77 | 0.69-0.87 | 1.21E-05 | 1613 | 1.00 | 0.86-1.17 | 9.86E-01 | 1.21E-05 | 0.77 | 0.89 | 0 |
| 3 | rs6794945 | 1.35E+08 | T | 3318 | 0.78 | 0.7-0.88 | 5.88E-05 | 1614 | 1.04 | 0.89-1.21 | 6.39E-01 | 6.09E-05 | 0.78 | 0.67 | 0 |
| 3 | rs7635876 | 1.58E+08 | T | 3318 | 1.39 | 1.19-1.63 | 4.18E-05 | 1615 | 1.01 | 0.78-1.31 | 9.51E-01 | 4.12E-05 | 1.39 | 0.92 | 0 |
| 3 | rs1842840 | 1.58E+08 | T | 3319 | 1.27 | 1.13-1.42 | 4.20E-05 | 1617 | 1.00 | 0.86-1.15 | 9.51E-01 | 4.16E-05 | 1.27 | 0.77 | 0 |
| 3 | rs11715321 | 1.58E+08 | C | 3318 | 1.27 | 1.14-1.43 | 3.35E-05 | 1618 | 1.00 | 0.87-1.16 | 9.85E-01 | 3.37E-05 | 1.27 | 0.84 | 0 |
| 4 | rs10009111 | 10580521 | G | 3318 | 0.76 | 0.68-0.85 | 1.35E-06 | 1616 | 0.90 | 0.78-1.03 | 1.37E-01 | 1.94E-06 | 0.77 | 0.19 | 42.15 |
| 4 | rs10011697 | 10580930 | G | 3319 | 0.76 | 0.68-0.85 | 1.50E-06 | 1616 | 0.90 | 0.78-1.03 | 1.37E-01 | 2.16E-06 | 0.77 | 0.19 | 42.04 |
| 4 | rs10516217 | 10582359 | A | 3319 | 1.26 | 1.13-1.4 | 4.57E-05 | 1615 | 0.90 | 0.78-1.05 | 1.72E-01 | 3.78E-05 | 1.26 | 0.53 | 0 |
| 4 | rs4487344 | 1.03E+08 | G | 3320 | 0.78 | 0.7-0.87 | 8.11E-06 | 1611 | 1.08 | 0.94-1.24 | 2.97E-01 | 1.23E-05 | 0.78 | 0.12 | 58.12 |
| 4 | rs13124455 | 1.03E+08 | A | 3318 | 1.36 | 1.19-1.54 | 4.00E-06 | 1613 | 0.94 | 0.79-1.11 | 4.47E-01 | 3.47E-06 | 1.36 | 0.63 | 0 |
| 4 | rs7669524 | 1.03E+08 | A | 3320 | 1.36 | 1.19-1.54 | 3.60E-06 | 1613 | 0.94 | 0.79-1.11 | 4.58E-01 | 3.15E-06 | 1.36 | 0.64 | 0 |
| 4 | rs768290 | 1.03E+08 | G | 3320 | 1.34 | 1.18-1.53 | 7.55E-06 | 1612 | 0.93 | 0.79-1.11 | 4.43E-01 | 6.58E-06 | 1.35 | 0.64 | 0 |
| 4 | rs12505043 | 1.03E+08 | T | 3319 | 0.78 | 0.68-0.89 | 1.79E-04 | 1614 | 1.05 | 0.89-1.23 | 5.76E-01 | 2.04E-04 | 0.78 | 0.4 | 0 |
| 4 | rs4482766 | 1.03E+08 | C | 3313 | 1.29 | 1.14-1.45 | 2.63E-05 | 1613 | 1.02 | 0.88-1.2 | 7.74E-01 | 2.52E-05 | 1.29 | 0.69 | 0 |
| 5 | rs106415 | 6845839 | A | 3320 | 0.8 | 0.71-0.89 | 5.96E-05 | 1614 | 0.91 | 0.8-1.05 | 1.98E-01 | 8.62E-05 | 0.8 | 0.16 | 48.95 |
| 5 | rs172384 | 36839982 | G | 3320 | 0.71 | 0.61-0.82 | 4.27E-06 | 1616 | 0.80 | 0.66-0.95 | 1.21E-02 | 1.51E-04 | 0.76 | 0 | 92.72 |
| 5 | rs292196 | 36949936 | T | 3320 | 0.7 | 0.6-0.81 | 4.21E-06 | 1614 | 0.82 | 0.68-0.98 | 2.59E-02 | 1.01E-04 | 0.75 | 0 | 91.75 |
| 5 | rs16903459 | 37067173 | G | 3317 | 0.72 | 0.62-0.84 | 4.16E-05 | 1616 | 0.83 | 0.69-1 | 5.40E-02 | 4.36E-04 | 0.76 | 0 | 89.38 |
| 5 | rs12658479 | 37242378 | C | 3313 | 0.76 | 0.66-0.87 | 8.61E-05 | 1614 | 0.89 | 0.75-1.06 | 1.82E-01 | 1.91E-04 | 0.77 | 0.06 | 72.4 |
| 5 | rs2460498 | 76177535 | A | 3320 | 0.76 | 0.67-0.87 | 5.71E-05 | 1613 | 0.88 | 0.73-1.07 | 2.13E-01 | 7.87E-05 | 0.77 | 0.22 | 33.48 |
| 6 | rs10948155 | 44795935 | C | 3319 | 0.79 | 0.7-0.89 | 6.47E-05 | 1616 | 1.02 | 0.88-1.19 | 7.68E-01 | 7.04E-05 | 0.79 | 0.5 | 0 |
| 6 | rs633668 | 1.69E+08 | A | 3320 | 1.31 | 1.16-1.49 | 2.22E-05 | 1618 | 0.96 | 0.82-1.13 | 6.55E-01 | 1.54E-05 | 1.32 | 0.41 | 0 |
| 8 | rs964307 | 1.1E+08 | G | 3320 | 0.77 | 0.68-0.86 | 1.05E-05 | 1616 | 0.92 | 0.79-1.06 | 2.49E-01 | 3.27E-05 | 0.78 | 0.02 | 81.5 |
| 8 | rs9297425 | 1.1E+08 | T | 3320 | 0.77 | 0.68-0.86 | 1.05E-05 | 1611 | 0.91 | 0.78-1.06 | 2.31E-01 | 3.21E-05 | 0.78 | 0.02 | 81.22 |
| 8 | rs7015262 | 1.11E+08 | G | 3320 | 0.78 | 0.7-0.88 | 3.60E-05 | 1612 | 0.93 | 0.81-1.08 | 3.68E-01 | 7.30E-05 | 0.79 | 0.06 | 71.64 |
| 9 | rs12683218 | 12411929 | G | 3318 | 1.33 | 1.17-1.51 | 1.29E-05 | 1612 | 0.97 | 0.83-1.14 | 7.11E-01 | 1.15E-05 | 1.33 | 0.64 | 0 |
| 9 | rs2289006 | 18768319 | T | 3320 | 0.76 | 0.68-0.86 | 4.41E-06 | 1609 | 1.01 | 0.87-1.16 | 9.36E-01 | 4.88E-06 | 0.77 | 0.48 | 0 |
| 9 | rs894520 | 38179527 | C | 3205 | 1.29 | 1.14-1.45 | 4.35E-05 | 1610 | 1.13 | 0.97-1.31 | 1.12E-01 | 2.86E-05 | 1.29 | 0.28 | 15.44 |
| 9 | rs10867228 | 80449015 | C | 3320 | 1.37 | 1.17-1.61 | 7.85E-05 | 1618 | 1.07 | 0.9-1.28 | 4.52E-01 | 7.22E-05 | 1.37 | 0.69 | 0 |
| 9 | rs10868564 | 89157591 | C | 3318 | 1.27 | 1.13-1.42 | 6.50E-05 | 1616 | 1.03 | 0.89-1.19 | 6.61E-01 | 6.16E-05 | 1.27 | 0.72 | 0 |
| 10 | rs13353058 | 1.25E+08 | G | 3317 | 1.62 | 1.34-1.96 | 5.26E-07 | 1617 | 1.01 | 0.8-1.26 | 9.55E-01 | 4.78E-07 | 1.62 | 0.76 | 0 |
| 11 | rs1255182 | 95112039 | T | 3319 | 0.8 | 0.72-0.89 | 6.56E-05 | 1614 | 0.89 | 0.77-1.03 | 1.07E-01 | 1.50E-04 | 0.81 | 0.04 | 76.97 |
| 11 | rs3748256 | 95161601 | G | 3319 | 0.79 | 0.71-0.89 | 6.81E-05 | 1615 | 0.89 | 0.78-1.03 | 1.15E-01 | 1.36E-04 | 0.8 | 0.06 | 72.15 |
| 11 | rs1784135 | 95171396 | A | 3319 | 0.79 | 0.71-0.89 | 7.90E-05 | 1609 | 0.88 | 0.77-1.02 | 8.24E-02 | 1.97E-04 | 0.81 | 0.03 | 78.94 |
| 11 | rs693364 | 95261574 | C | 3320 | 0.79 | 0.7-0.88 | 4.45E-05 | 1615 | 0.92 | 0.8-1.06 | 2.71E-01 | 6.71E-05 | 0.79 | 0.14 | 53.52 |
| 11 | rs10765777 | 95296033 | C | 3317 | 0.79 | 0.71-0.89 | 5.01E-05 | 1615 | 0.92 | 0.8-1.06 | 2.55E-01 | 7.84E-05 | 0.8 | 0.12 | 57.59 |
| 11 | rs3808977 | 95297409 | G | 3319 | 0.79 | 0.71-0.89 | 6.65E-05 | 1613 | 0.92 | 0.8-1.06 | 2.34E-01 | 1.08E-04 | 0.8 | 0.11 | 60.28 |
| 11 | rs11221390 | 1.28E+08 | T | 3320 | 0.75 | 0.65-0.86 | 2.85E-05 | 1613 | 0.93 | 0.77-1.12 | 4.40E-01 | 4.41E-05 | 0.75 | 0.16 | 48.23 |
| 12 | rs7308628 | 7403293 | A | 3264 | 1.74 | 1.32-2.3 | 8.67E-05 | 1618 | 0.73 | 0.55-0.98 | 3.36E-02 | 2.66E-05 | 1.77 | 0.66 | 0 |
| 12 | rs10431296 | 7478801 | C | 3319 | 1.64 | 1.29-2.08 | 5.67E-05 | 1618 | 0.82 | 0.59-1.14 | 2.32E-01 | 3.98E-05 | 1.64 | 0.7 | 0 |
| 12 | rs7961094 | 11803634 | T | 3317 | 1.34 | 1.16-1.54 | 7.69E-05 | 1614 | 0.89 | 0.73-1.07 | 2.17E-01 | 5.45E-05 | 1.34 | 0.45 | 0 |
| 12 | rs6539747 | 82337800 | C | 3317 | 1.3 | 1.15-1.47 | 4.22E-05 | 1612 | 1.05 | 0.9-1.23 | 5.12E-01 | 4.07E-05 | 1.3 | 0.87 | 0 |
| 12 | rs7964484 | 82373606 | G | 3318 | 1.3 | 1.15-1.46 | 2.36E-05 | 1615 | 1.03 | 0.88-1.19 | 7.51E-01 | 2.30E-05 | 1.3 | 0.98 | 0 |
| 13 | rs9533108 | 41922710 | C | 3317 | 0.78 | 0.7-0.88 | 1.72E-05 | 1615 | 1.13 | 0.98-1.31 | 8.43E-02 | 4.71E-05 | 0.79 | 0.01 | 83.88 |
| 13 | rs665657 | 41987378 | T | 3316 | 1.28 | 1.13-1.45 | 7.54E-05 | 1612 | 1.16 | 0.99-1.36 | 6.29E-02 | 3.33E-05 | 1.3 | 0.12 | 59.36 |
| 13 | rs990466 | 48218469 | G | 3320 | 0.77 | 0.68-0.87 | 5.66E-05 | 1613 | 1.21 | 1.03-1.42 | 2.11E-02 | 2.17E-04 | 0.79 | 0 | 87.65 |
| 13 | rs1164503 | 75765746 | A | 3320 | 1.29 | 1.14-1.46 | 3.34E-05 | 1615 | 1.09 | 0.95-1.26 | 2.31E-01 | 2.79E-05 | 1.29 | 0.5 | 0 |
| 13 | rs529041 | 1.1E+08 | A | 3320 | 1.54 | 1.26-1.88 | 1.99E-05 | 1614 | 0.83 | 0.63-1.09 | 1.73E-01 | 1.58E-05 | 1.55 | 0.69 | 0 |
| 13 | rs7995158 | 1.1E+08 | A | 3318 | 1.25 | 1.12-1.4 | 7.53E-05 | 1613 | 1.06 | 0.92-1.22 | 4.13E-01 | 6.36E-05 | 1.26 | 0.5 | 0 |
| 15 | rs7164857 | 91693946 | T | 3320 | 0.8 | 0.71-0.89 | 6.52E-05 | 1613 | 1.03 | 0.9-1.19 | 6.59E-01 | 6.53E-05 | 0.8 | 0.89 | 0 |
| 15 | rs17541566 | 91697940 | G | 3318 | 1.32 | 1.17-1.49 | 9.23E-06 | 1614 | 0.92 | 0.79-1.08 | 3.18E-01 | 7.49E-06 | 1.32 | 0.56 | 0 |
| 16 | rs9939858 | 9585398 | T | 3320 | 1.56 | 1.28-1.89 | 6.21E-06 | 1617 | 0.84 | 0.66-1.07 | 1.58E-01 | 1.17E-06 | 1.59 | 0.42 | 0 |
| 16 | rs407146 | 13223156 | T | 3319 | 1.26 | 1.12-1.41 | 7.06E-05 | 1614 | 1.00 | 0.86-1.16 | 9.77E-01 | 7.16E-05 | 1.26 | 0.98 | 0 |
| 16 | rs11647727 | 20263666 | A | 3319 | 0.72 | 0.63-0.82 | 7.03E-07 | 1613 | 0.92 | 0.79-1.07 | 2.80E-01 | 2.43E-06 | 0.73 | 0.02 | 80.29 |
| 16 | rs4506906 | 20264899 | C | 3320 | 0.79 | 0.7-0.88 | 4.76E-05 | 1614 | 0.90 | 0.78-1.04 | 1.39E-01 | 1.71E-04 | 0.8 | 0.01 | 84.35 |
| 16 | rs4293393 | 20272089 | C | 3320 | 0.67 | 0.58-0.78 | 1.45E-07 | 1614 | 0.93 | 0.77-1.11 | 4.00E-01 | 3.30E-07 | 0.68 | 0.08 | 67.53 |
| 16 | rs13333226 | 20273155 | G | 3319 | 0.67 | 0.58-0.78 | 1.14E-07 | 1615 | 0.91 | 0.76-1.08 | 2.82E-01 | 3.86E-07 | 0.68 | 0.03 | 77.71 |
| 16 | rs4496151 | 20280791 | T | 3320 | 0.79 | 0.7-0.88 | 4.13E-05 | 1608 | 0.91 | 0.79-1.05 | 1.84E-01 | 1.01E-04 | 0.8 | 0.03 | 77.8 |
| 18 | rs1942526 | 53769875 | G | 3318 | 1.72 | 1.31-2.25 | 8.46E-05 | 1616 | 0.91 | 0.72-1.16 | 4.61E-01 | 8.20E-05 | 1.72 | 0.9 | 0 |
| 18 | rs1893469 | 53776497 | G | 3320 | 1.65 | 1.31-2.08 | 2.48E-05 | 1617 | 0.99 | 0.8-1.24 | 9.46E-01 | 2.38E-05 | 1.65 | 0.86 | 0 |
| 19 | rs11880417 | 33022748 | G | 3320 | 0.79 | 0.71-0.89 | 3.76E-05 | 1614 | 1.03 | 0.89-1.19 | 6.67E-01 | 3.77E-05 | 0.79 | 0.87 | 0 |
| 19 | rs4804925 | 35515227 | G | 3319 | 0.73 | 0.63-0.84 | 3.04E-05 | 1613 | 1.04 | 0.88-1.23 | 6.40E-01 | 3.09E-05 | 0.73 | 0.78 | 0 |
| 19 | rs444816 | 49130206 | G | 3320 | 1.22 | 1.09-1.37 | 5.66E-04 | 1613 | 0.94 | 0.82-1.08 | 4.07E-01 | 4.83E-04 | 1.22 | 0.5 | 0 |
| 19 | rs381872 | 49157996 | A | 3320 | 1.26 | 1.13-1.41 | 5.73E-05 | 1613 | 0.94 | 0.82-1.09 | 4.21E-01 | 4.80E-05 | 1.26 | 0.49 | 0 |
| 19 | rs383133 | 49158936 | C | 3316 | 1.33 | 1.17-1.51 | 1.24E-05 | 1615 | 0.91 | 0.78-1.08 | 2.84E-01 | 7.93E-06 | 1.34 | 0.39 | 0 |
| 20 | rs2295179 | 8626446 | G | 3320 | 0.8 | 0.71-0.9 | 1.63E-04 | 1611 | 0.89 | 0.76-1.04 | 1.39E-01 | 3.44E-04 | 0.81 | 0.05 | 74.18 |
| 20 | rs8123323 | 8643581 | C | 3320 | 0.78 | 0.69-0.88 | 4.50E-05 | 1614 | 0.93 | 0.79-1.09 | 3.71E-01 | 5.98E-05 | 0.78 | 0.24 | 28.98 |
| 20 | rs172038 | 22309396 | G | 3317 | 0.6 | 0.47-0.77 | 5.92E-05 | 1617 | 0.95 | 0.74-1.21 | 6.53E-01 | 6.02E-05 | 0.6 | 0.85 | 0 |
| 20 | rs199843 | 22313546 | G | 3316 | 0.59 | 0.46-0.76 | 3.80E-05 | 1617 | 1.00 | 0.78-1.28 | 9.88E-01 | 4.69E-05 | 0.6 | 0.38 | 0 |
| 20 | rs6022204 | 51052745 | A | 3320 | 0.52 | 0.37-0.71 | 5.23E-05 | 1614 | 0.87 | 0.64-1.17 | 3.53E-01 | 8.26E-05 | 0.53 | 0.25 | 23.44 |
| 20 | rs2244665 | 53587166 | G | 3320 | 1.26 | 1.12-1.41 | 8.21E-05 | 1617 | 1.04 | 0.89-1.21 | 6.15E-01 | 8.13E-05 | 1.26 | 0.78 | 0 |
| 20 | rs682132 | 53605875 | G | 3317 | 0.77 | 0.69-0.86 | 5.32E-06 | 1593 | 1.01 | 0.87-1.16 | 9.19E-01 | 5.34E-06 | 0.77 | 0.98 | 0 |
| 20 | rs487331 | 53608771 | T | 3289 | 0.77 | 0.69-0.86 | 2.73E-06 | 1612 | 1.00 | 0.86-1.15 | 9.61E-01 | 2.76E-06 | 0.77 | 0.8 | 0 |
| 20 | rs555848 | 53613135 | C | 3315 | 0.77 | 0.69-0.86 | 3.48E-06 | 1615 | 1.04 | 0.9-1.2 | 6.40E-01 | 3.52E-06 | 0.77 | 0.79 | 0 |
