# Supplementary material for: Genome-Wide Association Study of Blood Pressure Extremes Identifies Variant near UMOD Associated with Hypertension
Source: PLoS Genet. 2010 Oct 28;6(10):e1001177. doi: 10.1371/journal.pgen.1001177 (PMC2965757; doi:10.1371/journal.pgen.1001177)
Supplement: Text S1 — Acknowledgments. (0.06 MB DOC) [file pgen.1001177.s005.doc]

**Acknowledgements**

**Institute of Cardiovascular and Medical Sciences, University of Glasgow.**

We are very grateful to CAMS staff who assisted QC of the discovery samples and review of cluster plots.

Adyani Md Redzuan, Aiste Monkeviciute, Angela Bradshaw, Annika Delles, Alan Parker, Caline Koh Tan, Carolyn Haggerty, Chiara Taurino, David Carty, Emily Ord, Fernando Martinez Garcia, Jenny Greig, Jenny McLachlan, Jim Mcculloch, John McClure, Kirsten Douglas, Laura Graham, Laura Denby, Laura Paul, Elizabeth Beattie, Lorraine Work, Ruth Mackenzie, Samantha Alvarez-Madrazo, Sandra MacDonald, Teresa, Ulf Neisius, Wendy Crawford, Weiling Sarah Li.

**Istituto Auxologico Italiano**

We are very grateful to Donatella Mihalich, Luisella Alberti, Raffaella Cancello, Alessandra Mihalich, Emanuela Ponti for their assistance.

**The Global BPgen Consortium**

Christopher Newton-Cheh^1,2,3^, Toby Johnson^4,5,6^, Vesela Gateva^7^, Martin D Tobin^8^, Murielle Bochud^5^, Lachlan Coin^9^, Samer S Najjar^10^, Jing Hua Zhao^11,12^, Simon C Heath^13^, Susana Eyheramendy^14,15^, Konstantinos Papadakis^16^, Benjamin F Voight^1,3^, Laura J Scott^7^, Feng Zhang^17^, Martin Farrall^18,19^, Toshiko Tanaka^20,21^, Chris Wallace^22,23^, John C Chambers^9^, Kay-Tee Khaw^12,24^, Peter Nilsson^25^, Pim van der Harst^26^, Silvia Polidoro^27^, Diederick E Grobbee^28^, N Charlotte Onland-Moret^28,29^, Michiel L Bots^28^, Louise V Wain^8^, Katherine S Elliott^19^, Alexander Teumer^30^, Jian'an Luan^11^, Gavin Lucas^31^, Johanna Kuusisto^32^, Paul R Burton^8^, David Hadley^16^, Wendy L McArdle^33^, Wellcome Trust Case Control Consortium^34^, Morris Brown^35^, Anna Dominiczak^36^, Stephen J Newhouse^22^, Nilesh J Samani^37^, John Webster^38^, Eleftheria Zeggini^19,39^, Jacques S Beckmann^4,40^, Sven Bergmann^4,6^, Noha Lim^41^, Kijoung Song^41^, Peter Vollenweider^42^, Gerard Waeber^42^, Dawn M Waterworth^41^, Xin Yuan^41^, Leif Groop^43,44^, Marju Orho-Melander^25^, Alessandra Allione^27^, Alessandra Di Gregorio^27,45^, Simonetta Guarrera^27^, Salvatore Panico^46^, Fulvio Ricceri^27^, Valeria Romanazzi^27,45^, Carlotta Sacerdote^47^, Paolo Vineis^9,27^, Inês Barroso^12,39^, Manjinder S Sandhu^11,12,24^, Robert N Luben^12,24^, Gabriel J. Crawford^3^, Pekka Jousilahti^48^, Markus Perola^48,49^, Michael Boehnke^7^, Lori L Bonnycastle^50^, Francis S Collins^50^, Anne U Jackson^7^, Karen L Mohlke^51^, Heather M Stringham^7^, Timo T Valle^52^, Cristen J Willer^7^, Richard N Bergman^53^, Mario A Morken^50^, Angela Döring^15^, Christian Gieger^15^, Thomas Illig^15^, Thomas Meitinger^54,55^, Elin Org^56^, Arne Pfeufer^54^, H Erich Wichmann^15,57^, Sekar Kathiresan^1,2,3^, Jaume Marrugat^31^, Christopher J O'Donnell^58,59^, Stephen M Schwartz^60,61^, David S Siscovick^60,61^, Isaac Subirana^31,62^, Nelson B Freimer^63^, Anna-Liisa Hartikainen^64^, Mark I McCarthy^19,65,66^, Paul F O’Reilly^9^, Leena Peltonen^39,49^, Anneli Pouta^64,67^, Paul E de Jong^68^, Harold Snieder^69^, Wiek H van Gilst^26^, Robert Clarke^70^, Anuj Goel^18,19^, Anders Hamsten^71^, John F Peden^18,19^, Udo Seedorf^72^, Ann-Christine Syvänen^73^, Giovanni Tognoni^74^, Edward G Lakatta^10^, Serena Sanna^75^, Paul Scheet^76^, David Schlessinger^77^, Angelo Scuteri^78^, Marcus Dörr^79^, Florian Ernst^30^, Stephan B Felix^79^, Georg Homuth^30^, Roberto Lorbeer^80^, Thorsten Reffelmann^79^, Rainer Rettig^81^, Uwe Völker^30^, Pilar Galan^82^, Ivo G Gut^13^, Serge Hercberg^82^, G Mark Lathrop^13^, Diana Zeleneka^13^, Panos Deloukas^12,39^, Nicole Soranzo^17,39^, Frances M Williams^17^, Guangju Zhai^17^, Veikko Salomaa^48^, Markku Laakso^32^, Roberto Elosua^31,62^, Nita G Forouhi^11^, Henry Völzke^80^, Cuno S Uiterwaal^28^, Yvonne T van der Schouw^28^, Mattijs E Numans^28^, Giuseppe Matullo^27,45^, Gerjan Navis^68^, Göran Berglund^25^, Sheila A Bingham^12,83^, Jaspal S Kooner^84^, John M Connell^36^, Stefania Bandinelli^85^, Luigi Ferrucci^21^, Hugh Watkins^18,19^, Tim D Spector^17^, Jaakko Tuomilehto^52,86,87^, David Altshuler^1,3,88,89^, David P Strachan^16^, Maris Laan^56^, Pierre Meneton^90^, Nicholas J Wareham^11,12^, Manuela Uda^75^, Marjo-Riitta Jarvelin^9,67,91^, Vincent Mooser^41^, Olle Melander^25^, Ruth JF Loos^11,12^, Paul Elliott^9^, Gonçalo R Abecasis^92^, Mark Caulfield^22^, Patricia B Munroe^22^

1. Center for Human Genetic Research, Massachusetts General Hospital, 185 Cambridge Street, Boston, MA 02114, USA

2. Cardiovascular Research Center, Massachusetts General Hospital, Boston, Massachusetts 02114, USA

3. Program in Medical and Population Genetics, Broad Institute of Harvard and Massachusetts Institute of Technology, Cambridge, Massachusetts, 02142, USA

4. Department of Medical Genetics, University of Lausanne, 1005 Lausanne, Switzerland

5. University Institute for Social and Preventative Medicine, Centre Hospitalier Universitaire Vaudois (CHUV) and University of Lausanne, 1005 Lausanne, Switzerland

6. Swiss Institute of Bioinformatics, Switzerland

7. Department of Biostatistics and Center for Statistical Genetics, University of Michigan, Ann Arbor, MI 48109, USA

8. Departments of Health Sciences & Genetics, Adrian Building, University of Leicester, University Road, Leicester LE1 7RH

9. Department of Epidemiology and Public Health, Imperial College London, St Mary's Campus, Norfolk Place, London W2 1PG, UK

10. Laboratory of Cardiovascular Science, Intramural Research Program, National Institute on Aging, National Institutes of Health, Baltimore, Maryland, USA 21224

11. MRC Epidemiology Unit, Institute of Metabolic Science, Addenbrooke's Hospital, Cambridge CB2 0QQ, UK

12. Cambridge - Genetics of Energy Metabolism (GEM) Consortium, Cambridge, UK

13. Centre National de Génotypage, 2 rue Gaston Crémieux, CP 5721, 91 057 Evry Cedex, France

14. Pontificia Universidad Catolica de Chile, Vicuña Mackenna 4860, Facultad de Matematicas, Casilla 306, Santiago 22, Chile, 7820436

15. Institute of Epidemiology, Helmholtz Zentrum München, German Research Centre for Environmental Health, 85764 Neuherberg, Germany

16. Division of Community Health Sciences, St George’s, University of London, London SW17 0RE, UK

17. Department of Twin Research & Genetic Epidemiology, King's College London, London SE1 7EH

18. Department of Cardiovascular Medicine, University of Oxford

19. The Wellcome Trust Centre for Human Genetics, Roosevelt Drive, Oxford, OX3 7BN, UK

20. Medstar Research Institute, 3001 S. Hanover Street, Baltimore, MD 21250, USA

21. Clinical Research Branch, National Institute on Aging, Baltimore, MD, 21250 USA

22. Clinical Pharmacology and The Genome Centre, William Harvey Research Institute, Barts and The London School of Medicine and Dentistry, Queen Mary University of London, London EC1M 6BQ

23. JDRF/WT Diabetes and Inflammation Laboratory, Cambridge Institute for Medical Research University of Cambridge, Wellcome Trust/MRC Building, Addenbrooke's Hospital Cambridge, CB2 0XY

24. Department of Public Health and Primary Care, Institute of Public Health, University of Cambridge, Cambridge CB2 2SR, UK

25. Department of Clinical Sciences, Lund University, Malmö University Hospital, SE-20502 Malmö, Sweden

26. Department of Cardiology University Medical Center Groningen, University of Groningen, Hanzeplein 1, 9700 RB Groningen, The Netherlands

27. ISI Foundation (Institute for Scientific Interchange), Villa Gualino, Torino, 10133, Italy

28. Julius Center for Health Sciences and Primary Care, University Medical Center Utrecht, STR 6.131, PO Box 85500, 3508 GA Utrecht, The Netherlands

29. Complex Genetics Section, Department of Medical Genetics - DBG, University Medical Center Utrecht, STR 2.2112, PO Box 85500, 3508 GA Utrecht, The Netherlands.

30. Interfaculty Institute for Genetics and Functional Genomics, Ernst-Moritz-Arndt-University Greifswald, 17487 Greifswald, Germany

31. Cardiovascular Epidemiology and Genetics, Institut Municipal d'Investigació Mèdica, Barcelona, Spain

32. Department of Medicine University of Kuopio 70210 Kuopio, Finland

33. ALSPAC Laboratory, Department of Social Medicine, University of Bristol, BS8 2BN, UK

34. A full list of authors is provided in the supplementary methods online.

35. Clinical Pharmacology Unit, University of Cambridge, Addenbrookes Hospital, Cambridge, UK CB2 2QQ

36. BHF Glasgow Cardiovascular Research Centre, University of Glasgow, Glasgow, UK G12 8TA

37. Department of Cardiovascular Science, University of Leicester, Glenfield Hospital, Groby Road, Leicester, LE3 9QP, UK

38. Aberdeen Royal Infirmary, Aberdeen, UK

39. Wellcome Trust Sanger Institute, Wellcome Trust Genome Campus, Hinxton, Cambridge CB10 1SA, UK

40. Service of Medical Genetics, Centre Hospitalier Universitaire Vaudois (CHUV), Lausanne, 1011, Switzerland

41. Genetics Division, GlaxoSmithKline, King of Prussia, PA 19406, USA

42. Department of Internal Medicine, Centre Hospitalier Universitaire Vaudois (CHUV) 1011 Lausanne, Switzerland

43. Department of Clinical Sciences, Diabetes and Endocrinology Research Unit, University Hospital, Malmö

44. Lund University, Malmö S-205 02, Sweden

45. Department of Genetics, Biology and Biochemistry, University of Torino, Torino, 10126, Italy

46. Department of Clinical and Experimental Medicine, Federico II University, Naples, 80100, Italy

47. Unit of Cancer Epidemiology, University of Turin and Centre for Cancer Epidemiology and Prevention (CPO Piemonte), Turin, 10126, Italy

48. National Institute for Welfare and Health P.O. Box 30, FI-00271 Helsinki, Finland

49. Institute for Molecular Medicine Finland FIMM, University of Helsinki and National Public Health Institute

50. Genome Technology Branch, National Human Genome Research Institute, Bethesda, MD 20892, USA

51. Department of Genetics, University of North Carolina, Chapel Hill, NC 27599, USA

52. Diabetes Unit, Department of Epidemiology and Health Promotion, National Public Health Institute, 00300 Helsinki, Finland

53. Physiology and Biophysics USC School of Medicine 1333 San Pablo Street, MMR 626 Los Angeles, California 90033

54. Institute of Human Genetics, Helmholtz Zentrum München, German Research Centre for Environmental Health, 85764 Neuherberg, Germany

55. Institute of Human Genetics, Technische Universität München, 81675 Munich, Germany

56. Institute of Molecular and Cell Biology, University of Tartu, 51010 Tartu, Estonia

57. Ludwig Maximilians University, IBE, Chair of Epidemiology, Munich

58. Cardiovascular Research Center and Cardiology Division, Massachusetts General Hospital, Boston, Massachusetts 02114, USA

59. Framingham Heart Study and National, Heart, Lung, and Blood Institute, Framingham, Massachusetts 01702, USA

60. Cardiovascular Health Research Unit, Departments of Medicine and Epidemiology, University of Washington, Seattle, Washington, 98101 USA

61. Department of Epidemiology, University of Washington, Seattle, Washington, 98195 USA

62. CIBER Epidemiología y Salud Pública, Barcelona, Spain

63. Center for Neurobehavioral Genetics, Gonda Center, Room 3506, 695 Charles E Young Drive South, Box 951761, UCLA, Los Angeles, CA 90095.

64. Department of Clinical Sciences/ Obstetrics and Gynecology, P.O. Box 5000 Fin-90014, University of Oulu, Finland

65. Oxford Centre for Diabetes, Endocrinology and Metabolism, University of Oxford, Churchill Hospital, Old Road, Headington, Oxford OX3 7LJ, UK

66. Oxford NIHR Biomedical Research Centre, Churchill Hospital, Old Road, Headington, Oxford, UK OX3 7LJ

67. Department of Child and Adolescent Health, National Public Health Institute (KTL), Aapistie 1, P.O. Box 310, FIN-90101 Oulu, Finland

68. Division of Nephrology, Department of Medicine University Medical Center Groningen, University of Groningen, Hanzeplein 1, 9700 RB Groningen, The Netherlands

69. Unit of Genetic Epidemiology and Bioinformatics, Department of Epidemiology University Medical Center Groningen, University of Groningen, Hanzeplein 1, 9700 RB Groningen, The Netherlands

70. Clinical Trial Service Unit and Epidemiological Studies Unit (CTSU), University of Oxford, Richard Doll Building, Roosevelt Drive, Oxford, OX3 7LF, UK

71. Atherosclerosis Research Unit, Department of Medicine Solna, Karolinska Institutet, Karolinska University Hospital Solna, Building L8:03, S-17176 Stockholm, Sweden

72. Leibniz-Institut für Arterioskleroseforschung an der Universität Münster, Domagkstr. 3, D-48149, Münster, Germany

73. Molecular Medicine, Department of Medical Sciences, Uppsala University, SE-751 85 Uppsala, Sweden

74. Consorzio Mario Negri Sud, Via Nazionale, 66030 Santa Maria Imbaro (Chieti), Italy

75. Istituto di Neurogenetica e Neurofarmacologia, CNR, Monserrato, 09042 Cagliari, Italy

76. Department of Epidemiology, Univ. of Texas M. D. Anderson Cancer Center, Houston, TX 77030

77. Laboratory of Genetics, Intramural Research Program, National Institute on Aging, National Institutes of Health, Baltimore, Maryland, USA 21224

78. Unitá Operativa Geriatria, Istituto Nazionale Ricovero e Cura per Anziani (INRCA) IRCCS, Rome, Italy

79. Department of Internal Medicine B, Ernst-Moritz-Arndt-University Greifswald, 17487 Greifswald, Germany

80. Institute for Community Medicine, Ernst-Moritz-Arndt-University Greifswald, 17487 Greifswald, Germany

81. Institute of Physiology, Ernst-Moritz-Arndt-University Greifswald, 17487 Greifswald, Germany

82. U557 Institut National de la Santé et de la Recherche Médicale, U1125 Institut National de la Recherche Agronomique, Université Paris 13, 74 rue Marcel Cachin, 93017 Bobigny Cedex, France

83. MRC Dunn Human Nutrition Unit, Wellcome Trust/MRC Building, Cambridge CB2 0XY, U.K

84. National Heart and Lung Institute, Imperial College London SW7 2AZ

85. Geriatric Rehabilitation Unit, Azienda Sanitaria Firenze (ASF), 50125, Florence, Italy

86. Department of Public Health, University of Helsinki, 00014 Helsinki, Finland

87. South Ostrobothnia Central Hospital, 60220 Seinäjoki, Finland

88. Department of Medicine and Department of Genetics, Harvard Medical School, Boston, Massachusetts 02115, USA

89. Diabetes Unit, Massachusetts General Hospital, Boston, Massachusetts 02114, USA

90. U872 Institut National de la Santé et de la Recherche Médicale, Faculté de Médecine Paris Descartes, 15 rue de l’Ecole de Médecine, 75270 Paris Cedex, France

91. Institute of Health Sciences and Biocenter Oulu, Aapistie 1, FIN-90101, University of Oulu, Finland

92. Center for Statistical Genetics, Department of Biostatistics, University of Michigan, Ann Arbor, Michigan 48109 USA

**PREVEND**

Investigators: Paul E de Jong, Gerjan Navis, Harold Snieder, Pim van

der Harst, Wiek H van Gilst. Coordination of genetic studies: G.N., W.H.v.G. Principal investigator: P.E.d.J. Study establishment: P.E.d.J., G.N., W.H.v.G. Data acquisition: P.E.d.J., G.N., P.v.d.H., W.H.v.G. Genotyping: P.v.d.H. Data analysis: H.S., P.v.d.H.

PREVEND genetics is supported by the Dutch Kidney Foundation (Grant E033), EU project grant GENECURE (FP-6 LSHM CT 2006 037697), and NWO VENI (grant number 916.76.170).

**KORA**

The KORA 500K blood pressure study was supported by Wellcome Trust International Senior Research Fellow (grant no. 070191/Z/03/Z) in Biomedical Science in Central Europe, by Alexander-von-Humboldt Foundation partnership (grant no. V-Fokoop-EST/1051368, V-Fokoop-1113183) and by Estonian Ministry of Education and Science core grant no. 0182721s06 (to M.Laan ). The KORA Augsburg studies were financed by the Helmholtz Zentrum München, German Research Center for Environmental Health, Neuherberg, Germany and supported by grants from the German Federal Ministry of Education and Research (BMBF). The KORA study group consists of H-E. Wichmann (speaker), A. Peters, C. Meisinger, T. Illig, R. Holle, J. John and co-workers who are responsible for the design and conduct of the KORA studies. Research on KORA was supported within the Munich Center of Health Sciences (MC Health) as part of LMUinnovativ and partially financed by the German National Genome Research Network (NGFN). Susana Eyheramendy Duerr is funded by Fondecyt, project number 11085012.

**B58C**

We acknowledge use of genotype data from the British 1958 Birth Cohort DNA collection, funded by the Medical Research Council grant G0000934 and the Wellcome Trust grant 068545/Z/02.

**Myocardial Infarction Genetics (MIGen) Consortium**

Investigators: Stephen M. Schwartz^1,2^, David S. Siscovick^1^, Jean Yee^1,2^, Yechiel Friedlander^3^, Roberto Elosua^4,5^, Jaume Marrugat^4^, Gavin Lucas^4^, Isaac Subirana^5,4^, Joan Sala^6^, Rafael Ramos^7^, Christopher J O'Donnell^8,9^, Sekar Kathiresan^10,11,12,8^, Calum A. MacRae^10,8^, Veikko Salomaa^13^, Aki S. Havulinna^13^, Leena Peltonen^12,14,15^, Olle Melander^16^, Goran Berglund^17^, Benjamin F. Voight^11,12,18^, Joel N. Hirschhorn^12,19^, Kiran Musunuru^10,11,12,8^, Mark Daly^11,12,8^, Shaun Purcell^11,12,20^, Aarti Surti^12^, Candace Guiducci^12^, Lauren Gianniny^12^, Daniel Mirel^12^, Melissa Parkin^12^, Noël Burtt^12^, Stacey Gabriel^12^, David Altshuler^11,12,8,18,19^.

Affiliations: 1. Cardiovascular Health Research Unit, Departments of Medicine and Epidemiology, University of Washington, Seattle, Washington, USA 2. Department of Epidemiology, University of Washington, Seattle, Washington, USA 3. Unit of Epidemiology, Hebrew University-Hadassah School of Public Health, Jerusalem, Israel 4. Cardiovascular Epidemiology and Genetics, Institut Municipal d'Investigacio Medica, Barcelona, Spain. 5. CIBER Epidemiología y Salud Pública, Barcelona, Spain. 6. Servei de Cardiologia i Unitat Coronària, Hospital de Girona Josep Trueta and Institut de Investigació Biomedica de Girona, Spain. 7. Unitat de Recerca i Unitat Docent de Medicina de Familia de Girona, IDIAP Jordi Gol, Institut Català de la Salut, Spain. 8. Department of Medicine, Harvard Medical School, Boston, Massachusetts 02115, USA. 9. Framingham Heart Study and National, Heart, Lung, and Blood Institute, Framingham, Massachusetts, 01702, USA 10. Cardiovascular Research Center and Cardiology Division, Massachusetts General Hospital, Boston, Massachusetts 02114, USA 11. Center for Human Genetic Research, Massachusetts General Hospital, Boston, Massachusetts 02114, USA 12. Program in Medical and Population Genetics, Broad Institute of Harvard and Massachusetts Institute of Technology, Cambridge, Massachusetts 02142, USA. 13. Department of Chronic Disease Prevention, THL-National Institute for Health and Welfare, Helsinki, Finland. 14. Wellcome Trust Sanger Institute, Cambridge CB10 1SA, UK. 15. Institute for Molecular Medicine, University of Helsinki, Helsinki 00029, Finland.

16. Department of Clinical Sciences, Hypertension and Cardiovascular Diseases, University Hospital, Malmö, Lund University, Malmö 20502, Sweden. 17. Department of Clinical Sciences, Internal Medicine, University Hospital Malmö, Lund University, Malmö 20502, Sweden. 18. Department of Molecular Biology, Massachusetts General Hospital, Boston, Massachusetts, 02114, USA. 19. Department of Genetics, Harvard Medical School, Boston, Massachusetts 02115, USA. 20. Stanley Center for Psychiatric Research, Broad Institute of MIT and Harvard, Cambridge, Massachusetts, 02142, USA.

The MIGen study was funded by the U.S. National Institutes of Health (NIH) and National Heart, Lung, and Blood Institute's STAMPEED genomics research program (R01 HL087676). Genotyping was partially funded by The Broad Institute Center for Genotyping and Analysis, which is supported by grant from the National Center for Research Resources (U54 RR020278). There was also partial funding from the US NIH (R01HL056931, P30ES007033, N01-HD-1-3107) (D.S.).

G.L. was supported by the Juan de la Cierva Program (JCI-2009_04684). S.K. is supported by a Doris Duke Charitable Foundation Clinical Scientist Development Award, a charitable gift from the Fannie E. Rippel Foundation, the Donovan Family Foundation, a career development award from the NIH, and institutional support from the Department of Medicine and Cardiovascular Research Center at Massachusetts General Hospital. V.S. was supported by the Sigrid Juselius Foundation and the Finnish Foundation for Cardiovascular Research.

This analysis was partially funded by the Ministerio de Ciencia e Innovación, Instituto de Salud Carlos III/FEDER (RD06/0009, PI061254, CIBERESP), AGAUR (SGR 2005/00577), and Fundacio Marato TV3.

**SHIP**

SHIP is part of the Community Medicine Research net of the University of Greifswald, Germany, which is funded by the Federal Ministry of Education and Research (grants no. 01ZZ9603, 01ZZ0103, and 01ZZ0403), the Ministry of Cultural Affairs as well as the Social Ministry of the Federal State of Mecklenburg-West Pomerania. Genome-wide data have been supported by the Federal Ministry of Education and Research (grant no. 03ZIK012) and a joint grant from Siemens Healthcare, Erlangen, Germany and the Federal State of Mecklenburg- West Pomerania. The University of Greifswald is a member of the ‘Center of Knowledge Interchange’ program of the Siemens AG.

**HERCULES**

The study was supported by the Swiss National Science Foundation (PROSPER 3200BO-111362/1, 3233BO-111361/1 to Murielle Bochud, FN-320000-116364 to Michel Burnier) and by the Swiss School of Public Health Plus (SSPH+).

**CoLaus**

We thank Vincent Mooser, Gérard Waeber, Sven Bergmann and Jacques Beckmann for their contribution to the CoLaus study. M.Bochud was supported by the Swiss National Science Foundation (PROSPER 3200BO-111362/1, 3233BO-111361/1) and by the Swiss School of Public Health Plus (SSPH+). P.Vollenweider received financial support from GlaxoSmithKline to build the CoLaus study. This work has been supported by GlaxoSmithKline, the Swiss National Science foundation (33CSO-122661) and the Faculty of Biology and Medicine of Lausanne, Switzerland.

**MONICA/BRIANZA Research Group:** Giancarlo Cesana, Marco Ferrario, Paolo Brambilla, Stefano Signorini, Roberto Sega, Cristina Menni and Lombardy Health Directorate.

**PAMELA Research Group:** Giuseppe Mancia, Guido Grassi, Cristina Giannattasio, Roberto Sega, Michele Bombelli, Rita Facchetti, Alberto Zanchetti.

**BRIGHT**

We would like to thank Philip Howard, Abiodun Onipinla, Charles Mein, Richard Dobson, and the Barts Genome Centre staff for assistance with genotyping. The BRIGHT study is extremely grateful to all the patients who participated in the study and the BRIGHT nursing team. The BRIGHT study principal investigators include Prof MJ Caulfield, Prof PB Munroe, Prof M Brown, Prof M Farrall, Prof NJ Samani, Prof AF Dominiczak, Prof JMC Connell, Prof D Clayton, Prof M Lathrop and Prof J Webster. The BRIGHT study is supported by the Medical Research Council of Great Britain (G9521010D) and the British Heart Foundation (PG/02/128). Prof A.F.D. and N.J.S. are supported by chairs funded by the British Heart Foundation. Toby Johnson was supported through a VIP award from the Wellcome Trust to Queen Mary University of London in the 2009/2010 academic year.

**ASCOT**

We thank all ASCOT trial participants, physicians, nurses, and practices in the participating countries for their important contribution to the study. In particular we thank Clare Muckian and David Toomey for their help in DNA extraction, storage, and handling. The ASCOT study and the collection of the ASCOT DNA repository were supported by Pfizer, New York, NY, USA. Funding for the ASCOT study was also provided by Servier Research Group, Paris, France, and Leo Laboratories, Copenhagen, Denmark.

**MPP and MDC**

These studies were supported by grants from the Swedish Medical Research Council, the Swedish Heart and Lung Foundation, the Medical Faculty of Lund University, Malmö University Hospital, the Albert Påhlsson Research Foundation, the Crafoord foundation, the Ernhold Lundströms Research Foundation, the Region Skane, the Hulda and Conrad Mossfelt Foundation, the King Gustaf V and Queen Victoria Foundation, the Lennart Hanssons Memorial Fund, and the Marianne and Marcus Wallenberg Foundation.

**NORDIL**

The NORDIL study was supported by a grant from Pharmacia.

**TwinsUK**

The study was funded by the Wellcome Trust, Arthritis Research Campaign, European Community’s Seventh Framework Programme (FP7/2007-2013)/grant agreement HEALTH-F2-2008-201865-GEFOS and EC Framework 7programme grant 200800 Treat OA /(FP7/2007-2013), ENGAGE project grant agreement HEALTH-F4-2007-201413 and the FP-5 GenomEUtwin Project (QLG2-CT-2002-01254). The study also receives support from the Dept of Health via the National Institute for Health Research (NIHR) comprehensive Biomedical Research Centre award to Guy's & St Thomas' NHS Foundation Trust in partnership with King's College London. TDS is an NIHR senior Investigator. The project also received support from a Biotechnology and Biological Sciences Research Council (BBSRC) project grant. (G20234) .The authors acknowledge the funding and support of the National Eye Institute via an NIH/CIDR genotyping project (PI: Terri Young). We thank the staff from the Genotyping Facilities at the Wellcome Trust Sanger Institute for sample preparation, Quality Control and Genotyping led by Leena Peltonen and Panos Deloukas; Le Centre National de Génotypage, France, led by Mark Lathrop, for genotyping; Duke University, North Carolina, USA, led by David Goldstein, for genotyping; and the Finnish Institute of Molecular Medicine, Finnish Genome Centre, University of Helsinki, led by Aarno Palotie. Genotyping was also performed by CIDR as part of an NEI/NIH project grant.

**Diabetes Genetics Initiative (DGI)**

See <Saxena et al. Science. 2007 Jun 1;316(5829):1331-6. Epub 2007 Apr 26> for full list of investigators and description of overall design. Investigators for the current project: Pankaj Arora, Olle Melander, Christopher Newton-Cheh.

We thank the participants of Sweden and Finland who contributed to the DGI study. This work was supported by the Novartis Institute for Biomedical Research. C.N.-C. is supported by a K23 (NIH HL80025), a Doris Duke Charitable Foundation Clinical Scientist Development Award, a Burroughs Wellcome Fund Career Award for Medical Scientists and institutional support from the Massachusetts General Hospital Cardiovascular Research Center and the Department of Medicine. O.M. is supported by the Swedish Medical Research Council, the Swedish Heart and Lung Foundation, the Medical Faculty of Lund University, Malmö University Hospital, the Albert Påhlsson Research Foundation, the Crafoord foundation, the Ernhold Lundströms Research Foundation, the Region Skane, the Hulda and Conrad Mossfelt Foundation, the King Gustaf V and Queen Victoria Foundation and the Lennart Hanssons Memorial Fund.

**Fenland**

We are grateful to all the volunteers for their time and help, and to the General Practitioners and practice staff for help with recruitment. We thank the Fenland Study Co-ordination team, the Field Epidemiology team and the Fenland Study Investigators.

The albuminuria measurement was performed by the Department of Clinical Biochemistry and Immunology. The Fenland Study is funded by the Wellcome Trust and the Medical Research Council.

**NESDA:** The infrastructure for the NESDA study is funded through the Geestkracht programme of the Dutch Scientific Organization (ZON-MW, grant number 10-000-1002) and matching funds from participating universities and mental health care organizations. Genotyping in NESDA was funded by the Genetic Association Information Network (GAIN) of the Foundation for the US National Institutes of Health.
